# Bayesian Convolutional Neural Networks for Uncertainty-Aware Classification of Infrasound Events

**DOI:** 10.3390/s26154955

**Published:** 2026-08-05

**Authors:** Hao Yin, Kai Zhang, Yu Lu, Yunfen Chang, Yunhui Wu, Fan Yang, Xuexu Li, Jiaoheng Xu, Xinliang Pang

**Affiliations:** Key Laboratory of Chemistry for NBC Hazards Protection, Beijing 102205, China; yinhao1207@163.com (H.Y.); jiudb@163.com (K.Z.); geolyu@163.com (Y.L.); yunshun0330@163.com (Y.C.); wuyunhui@sklnbcpc.cn (Y.W.); yangfan3132021@126.com (F.Y.); 202312490035@nuist.edu.cn (X.L.); xh304033@gmail.com (J.X.)

**Keywords:** infrasound signal classification, Bayesian convolutional neural networks, uncertainty quantification

## Abstract

**Highlights:**

**What are the main findings?**

- A Bayesian CNN framework is proposed for imbalanced infrasound signal classification without the need for data augmentation.
- The custom 4Conv3Fc architecture, trained via Bayesian inference, achieves a classification accuracy of 99.14%.
- The framework is model-agnostic and substantially improves the performance of baseline models such as LeNet-5 and AlexNet.

**What are the implications of the main findings?**

- The paper explores Bayesian CNN-based uncertainty quantification for multi-class infrasound classification and demonstrates its potential on the authors’ dataset.
- To our knowledge, it represents the first application of such joint uncertainty quantification within a Bayesian CNN to infrasound signal classification.

**Abstract:**

Accurate classification of infrasound signals is essential for nuclear-test verification, natural-hazard warning, and geophysical monitoring. Conventional convolutional neural networks (CNN) applied to this task tend to overfit small, class-imbalanced datasets and cannot quantify predictive uncertainty. To address these limitations, we introduce a Bayesian CNN framework that treats network weights as probability distributions and performs inference by variational approximation. LeNet-5, AlexNet, and 4Conv3Fc network serve as baselines and are converted into Bayes LeNet-5, Bayes AlexNet, and Bayes 4Conv3Fc. The short-time Fourier transform (STFT) provides time–frequency spectrograms as model input. On a highly imbalanced dataset comprising nuclear tests, chemical explosions, volcanic eruptions, rocket launches, earthquakes, and lightning, Bayes 4Conv3Fc reaches an overall accuracy of 99.14% without any data augmentation. Relative to the deterministic baselines, precision, recall, and F1-score increase by up to 6.91, 7.12, and 7.20 percentage points, respectively, and Cohen’s Kappa coefficient by up to 8.86 percentage points. Against class-weighted cross-entropy, a standard imbalance-handling baseline, the Bayesian models yield consistently lower Brier scores, indicating that the gains stem from principled uncertainty modelling rather than loss re-weighting alone.. This study quantifies both epistemic and aleatoric uncertainty in an infrasound signal classification model, and the calibration analysis validates that these uncertainty estimates are reliable, providing a basis for evaluating prediction reliability and diagnosing potential failure modes, thereby contributing to improved model interpretability. Coupled with an event-level data partitioning strategy, the evaluation faithfully reflects the model’s generalization to unseen events and offers a promising direction toward uncertainty-aware infrasound monitoring.

## 1. Introduction

Infrasound refers to acoustic waves with frequencies below 20 Hz, characterized by low frequency, low attenuation, long propagation distance, and strong penetration capability [1,2]. Its sources are extremely diverse: natural phenomena and human activities such as earthquakes [3], lightning [4,5,6], volcanic eruptions [7,8], nuclear tests [9,10,11], chemical explosions [12,13,14,15], and rocket launches [16,17,18] all generate infrasound signals. Benefiting from its low-frequency characteristics, infrasound can propagate over long distances via atmospheric waveguides, making it valuable for applications such as nuclear test monitoring, natural disaster warning, and geophysical research. However, long-distance propagation also makes infrasound signals inevitably subject to substantial influences from wind turbulence, atmospheric temperature, and terrain, causing waveform distortion and directly increasing the difficulty for classification models to generalize under varying environmental conditions. Therefore, sustained research on infrasound signal classification is the foundation for overcoming these limitations and building an automated, high-confidence global infrasound monitoring network, which is of both theoretical and practical importance.

Signal classification plays a crucial role in infrasound monitoring systems. Traditional infrasound signal classification methods primarily involve two key steps: acoustic feature extraction and classifier design. The essence of infrasound signal classification is to identify key features representing specific infrasound events. In previous studies, Mel-frequency cepstral coefficients [19,20], wavelet transforms [21,22,23], information spectral entropy [24,25], the Hilbert–Huang transform [26], and others have been used for infrasound signal representation. Regarding classifiers, support vector machines, Bayesian classifiers, and random forests are commonly employed for infrasound signal classification, alongside simpler algorithms such as k-nearest neighbours and decision trees [21,22,24,25,26,27,28]. Moreover, the effectiveness of artificial neural networks [22,23,25,29,30,31] as classifiers has also been validated. In most cases, support vector machines generally converge more reliably and achieve higher recognition accuracy. Although the traditional “feature extraction + classifier” approach can achieve high classification accuracy, it requires manually designed filters for noise reduction or additional feature extraction operations.

In recent years, deep learning technology has achieved breakthroughs in image, speech, and time-series signal processing, and infrasound event classification techniques have also evolved from traditional pattern recognition to today’s popular end-to-end deep learning models. The core advantage of such methods lies in their ability to automatically learn hierarchical discriminative features from raw data or simple transformations. For instance, Solomon et al. omitted the manual feature extraction step and employed deep neural networks, self-normalizing neural networks, fully convolutional neural networks, and long short-term memory (LSTM) networks to classify four types of infrasound signals from the Library of Typical Infrasonic Signals (LOTIS) dataset. Their experimental results verified the potential of deep neural network architectures for infrasound classification; however, the LSTM network only achieved a classification accuracy of 67% [32]. Subsequently, they also utilized the VGG model to recognize raw and preprocessed (bandpass-filtered and resampled) time-series signals, finding that the recognition accuracy for preprocessed signals was lower than that for raw signals [33]. Smith et al. directly used raw signals and two-dimensional spectrograms as inputs for deep neural networks (DNN) and convolutional neural networks (CNN), respectively, and their results showed that this approach has advantages in real-time performance and reduced computational complexity [23]. To address the scarcity of infrasound data, Wu et al. employed generative adversarial networks (GAN) to generate synthetic infrasound signals to augment the dataset and fed time–frequency spectrogram into a CNN, achieving good recognition performance [34].

Building on this, researchers have begun exploring more refined feature representations and network architectures to address the unique challenges of infrasound signals. Witsil et al. synthesized infrasound data based on physical models to train artificial neural networks (ANN) and temporal convolution neural networks (TCN) for distinguishing infrasound event signals from non-infrasound event signals, with ANN and TCN achieving recognition accuracies of 90% and 97%, respectively [35]. Bishop et al. developed a deep learning method that integrates infrasound detection and classification, directly feeding raw waveform data into a CNN with a self-attention layer; the accuracy for discriminating noise from non-noise data exceeded 96%, but the results were inconsistent when distinguishing stationary from non-stationary signals [36]. Leng et al. converted infrasound signals into two-dimensional time–frequency spectrogram and designed a model based on an improved LeNet-5 network, achieving a recognition accuracy of 84.1% for five common types of infrasound data [37]. Tan et al. focused on signal decomposition and multi-channel feature combination, proposing a method based on a multi-channel multi-scale convolutional attention network (MCMS-CBAMNet) [38]. Through CEEMDAN decomposition and IMF component selection, they effectively mitigated signal aliasing caused by multipath effects, achieving an average precision of 82.76% in chemical explosion and earthquake classification tasks. To further enhance the joint representation of spatiotemporal features, Tan et al. subsequently proposed a Parallel Convolutional Kernel, CBAM, and LSTM Network (PCMLN) model and a GAF-ConvLSTM-based framework. By combining signal assembly, two-dimensional GAF transformation, and convolutional LSTM networks, they elevated the classification accuracy for chemical explosions and earthquakes to 83.9% and 92.4% in two studies in 2025, respectively [39,40]. These studies demonstrate that hybrid deep models incorporating advanced signal processing techniques (e.g., empirical mode decomposition and Gramian angular fields) can more effectively mine the discriminative information hidden in infrasound signals.

Given the difficulty of acquiring infrasound data and the resulting small sample sizes, data augmentation and few-shot learning have become another research hotspot. Li et al. proposed a multi-scale SE-CNN-BiLSTM (squeeze excitation–convolutional neural network–bidirectional long short-term memory network) fusion model that automatically extracts spatiotemporal features of signals, achieving a classification accuracy of over 98% for chemical explosion and earthquake events [41]. Tan et al. addressed the overfitting problem in small-sample scenarios by proposing a method based on mixed virtual infrasound data augmentation (MVIDA) and a multiscale squeeze-and-excitation ResNet (MS-SE-ResNet), achieving an average classification accuracy of 81.12% on the augmented dataset [42]. Lu et al. extended this line of work, proposing a prediction model combining TimeGAN and a coordinated attention prototype network (CAPN), termed TimeGAN-CAPN. This method uses a temporal generative adversarial network to generate high-quality synthetic data in the latent space, effectively augmenting the small-sample dataset, and combines it with the dual-view mutual learning mechanism of CAPN, achieving performance markedly superior to traditional methods in a three-category classification task involving earthquakes, tsunamis, and volcanoes [43]. Zhao et al. combined LSTM with prototypical metrics and proposed a classification method suitable for variable-duration infrasound signals, attaining average recognition accuracies of 97.96% and 95.36% on public and measured datasets, respectively [44]. Yin et al. proposed a method based on short-time Fourier transform (STFT) and CNN, achieving an average recognition accuracy of 96.58% for six types of raw infrasound signals [45].

The above research indicates that deep learning methods represented by CNN, with their powerful automatic feature extraction capability and end-to-end learning mechanism, have been widely applied in infrasound signal classification and have achieved remarkable results. However, applying traditional deterministic neural networks to infrasound signal classification still presents inherent deficiencies. First, CNN typically require large amounts of data for effective training, whereas certain types of infrasound signals (e.g., nuclear tests) have become extremely rare since the signing of the Comprehensive Nuclear-Test-Ban Treaty, and high-quality labeled data are very limited [46]. Second, on small-sample datasets, CNN are prone to overfitting, resulting in poor generalization capability [42]. Although methods such as data augmentation, generative adversarial networks, and prototype networks mentioned in the above studies partially alleviate this problem, they mostly still aim to learn an optimal “point estimate” of weights, lacking the modeling of the model’s own confidence. Third, traditional CNN cannot quantify model uncertainty; their output is a deterministic prediction that cannot reflect the confidence of the prediction. In practical monitoring applications, especially in high-stakes decision-making scenarios such as nuclear test identification and natural disaster early warning, an overconfident erroneous prediction could lead to severe consequences. In contrast, a model capable of providing predictive uncertainty can offer analysts richer decision-making information, identifying boundary samples that require manual review.

To further improve classification accuracy under limited sample conditions, enhance model generalization ability, and fundamentally meet the practical demands for predictive reliability in infrasound signal classification, this paper proposes an infrasound signal classification method based on a Bayesian Convolutional Neural Network (Bayesian CNN). Compared with traditional CNN, this method introduces Bayesian statistical inference, assigning probability distributions rather than point estimates to network weights. This inherently provides a regularization effect, thereby more effectively alleviating overfitting problems in small-sample scenarios and demonstrating better generalization performance. Simultaneously, the Bayesian CNN can quantify predictive uncertainty (including epistemic uncertainty and aleatoric uncertainty), providing valuable confidence information for the decision-making process, thus enhancing model interpretability and decision reliability.

## 2. Materials and Methods

### 2.1. Data and Preprocessing

In this section, we first briefly introduce the infrasound signal dataset. We then explain why data augmentation is not employed in the experimental validation. Finally, we perform the necessary data interpolation and time–frequency analysis preprocessing on the dataset, explaining the reasons for adopting shape-preserving piecewise cubic interpolation and the short-time Fourier transform method.

#### 2.1.1. Infrasound Signal Datasets

**Figure 1:**
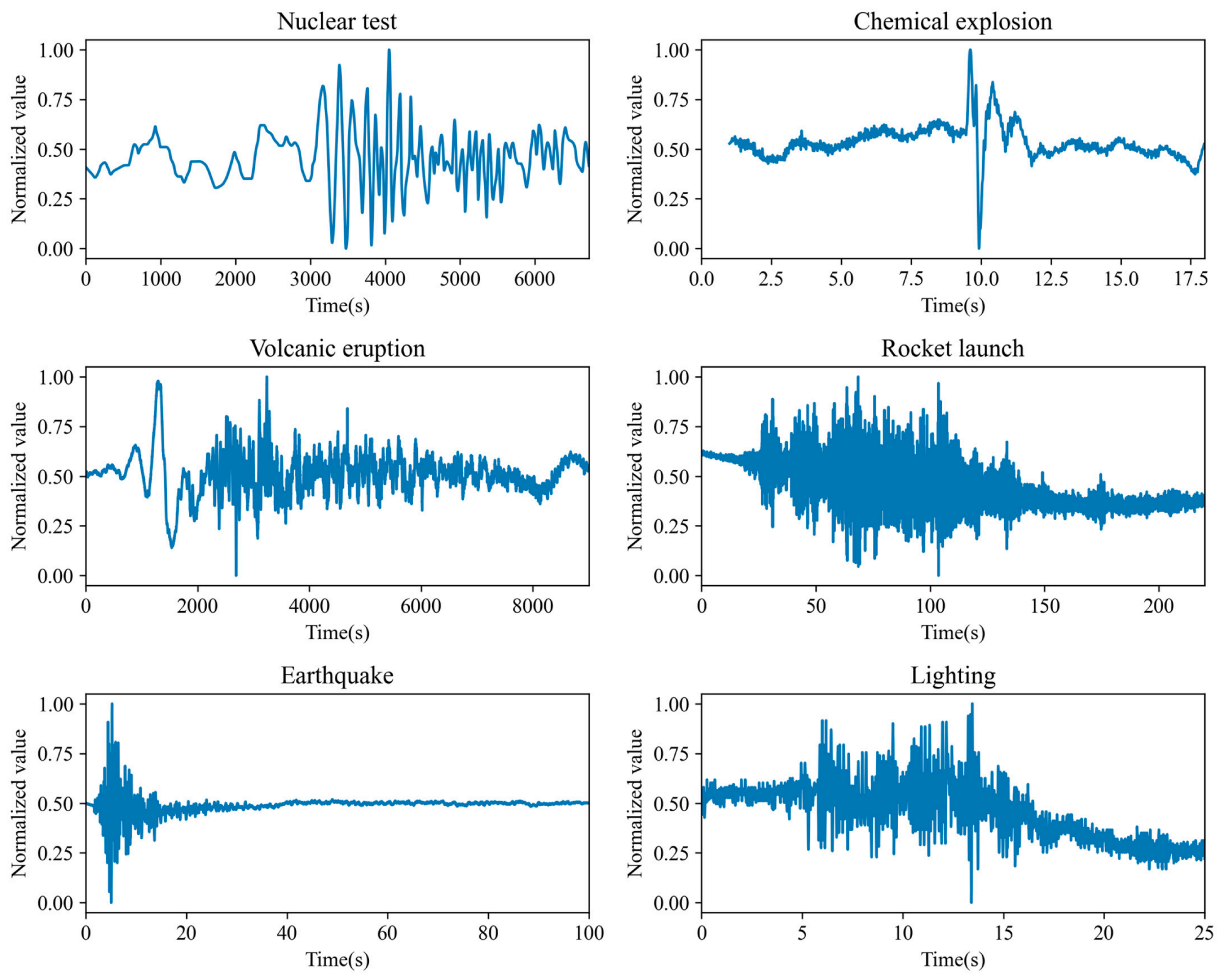
Time-domain waveform of six types of infrasound signal.

The infrasound data used in this study were obtained from multiple infrasound arrays of the International Monitoring System (IMS) and from actual observations recorded by infrasound monitoring stations deployed by our laboratory, involving a total of 96 fixed stations. The sensors predominantly consisted of MB3a (Seismo Wave, Rospez, France), Hyperion 5313A (Hyperion, Tupelo, MS, USA), MB2000 (Seismo Wave, Rospez, France), and MB2005 (Seismo Wave, Rospez, France) microbarometers. The recording period spans from October 1952 to January 2022, covering propagation paths under different seasons and a variety of atmospheric conditions. The source-to-receiver distances range from 34 km to 16,180 km, nearly encompassing propagation scales from local to global. The original sampling rates were 20, 40, 50, and 100 Hz. The duration of different signals varies considerably, from as short as 10 s to as long as several hours. Event-type labels were primarily assigned based on the Reviewed Event Bulletin (REB) issued by the International Data Centre (IDC) and the associated seismo-acoustic joint bulletin and were manually verified by trained analysts according to waveform characteristics, arrival times, and spectral information to ensure the reliability of the annotations. The dataset comprises six event types—nuclear tests, chemical explosions, volcanic eruptions, rocket launches, earthquakes, and lightning—with a total of 595 infrasound events and 2306 infrasound waveform recordings; detailed statistics are provided in Table 1. A representative time-series waveform for all six event types is illustrated in Figure 1.

It should be particularly noted that the 257 signals in the nuclear test category originate from only 96 independent nuclear test events (i.e., the same event recorded by multiple stations), and the 33 signals in the rocket launch category originate from 28 independent launch events. This indicates a strong event dependence among multiple records of the same event. If the data partitioning strategy does not control for this dependence, different records of the same event may appear in both the training and test sets, causing the model to memorize event-specific spectral features or propagation path characteristics and thereby severely overestimate the classification performance. To avoid such data leakage, this study strictly adopts an event-level stratified partitioning strategy, ensuring that all records belonging to the same event are assigned to only a single subset. This minimizes the data correlation among the training, validation, and test sets, allowing a more objective evaluation of the model’s generalization ability to unseen events.

For datasets with highly imbalanced classes, previous studies have developed a variety of methods to address this issue, as summarized by Buda et al. [47]. Existing research frameworks commonly rely on data augmentation techniques to achieve class balance in multi-class scenarios. However, this reliance necessitates carefully tracking the training and test sets before and after augmentation to ensure model reliability, which places higher demands on the rigor of experimental design. Using raw data not only fully preserves the statistical distribution characteristics of infrasound signals in real physical environments but also effectively captures the coupling characteristics between infrasound propagation and complex environmental factors such as atmospheric disturbances and terrain diffraction. Classification models trained on raw data can establish more robust physics–data associations during feature representation learning, which plays a crucial role in enhancing model generalization. Moreover, with the advancement of infrasound monitoring technology, continuously accumulated measured signals will provide valuable supplements to the dataset and offer a reliable foundation for incremental learning during model iteration. The method proposed in this paper not only circumvents the risk of distribution shift between synthetic and measured data but also effectively leverages the multi-scale features of infrasound signals in the time–frequency domain, thereby providing a new technical pathway for improving the classification performance of imbalanced infrasound signals.

#### 2.1.2. Data Preprocessing

Data acquired through the infrasound monitoring array originate from different observation equipment, and substantial differences exist in key parameters such as sampling rate and amplitude scale. Some nuclear explosion infrasound data were acquired through non-uniform sampling, resulting in inconsistent time intervals. In addition, the sampling rates of the remaining raw infrasound data—such as those from chemical explosions, volcanic eruptions, earthquakes, and lightning—also differ (e.g., 20 Hz, 40 Hz, 50 Hz, and 100 Hz). To unify the sampling frequency, interpolation methods are used to convert infrasound signals with different sampling rates into signals with a uniform sampling rate of 100 Hz. Both shape-preserving piecewise cubic interpolation and cubic spline interpolation possess good convergence and stability, as well as a certain degree of smoothness, and are of great significance in both theory and application. Following [45], we adopt the shape-preserving piecewise cubic interpolation method to interpolate the infrasound data, because the signal after shape-preserving piecewise cubic interpolation is relatively smooth, maintains the convexity requirement of the signal itself, and better conforms to the characteristics of actual signals. At the same time maximum-amplitude normalization is applied to eliminate inconsistencies among samples caused by differences in dimension or order of magnitude.

To enable the model to effectively learn the characteristic information of infrasound signals from both the time and frequency domains, this study employs the Short-Time Fourier Transform (STFT) to convert time-series signals into spectrograms. As a classical time–frequency analysis method, the STFT [48,49] segments a relatively long signal into shorter segments of equal length and computes the Fourier transform on each segment separately. By striking a compromise between time resolution and frequency resolution, the STFT can effectively reveal the time-varying spectral characteristics of infrasound signals and is suitable for analyzing various types of non-stationary infrasonic events. Moreover, compared with other time–frequency analysis methods such as the wavelet transform (WT) [50,51] and the Hilbert–Huang transform (HHT) [52], the STFT is computationally simpler and more straightforward. Therefore, it is reasonable to use the STFT in this study.

**Figure 2:**
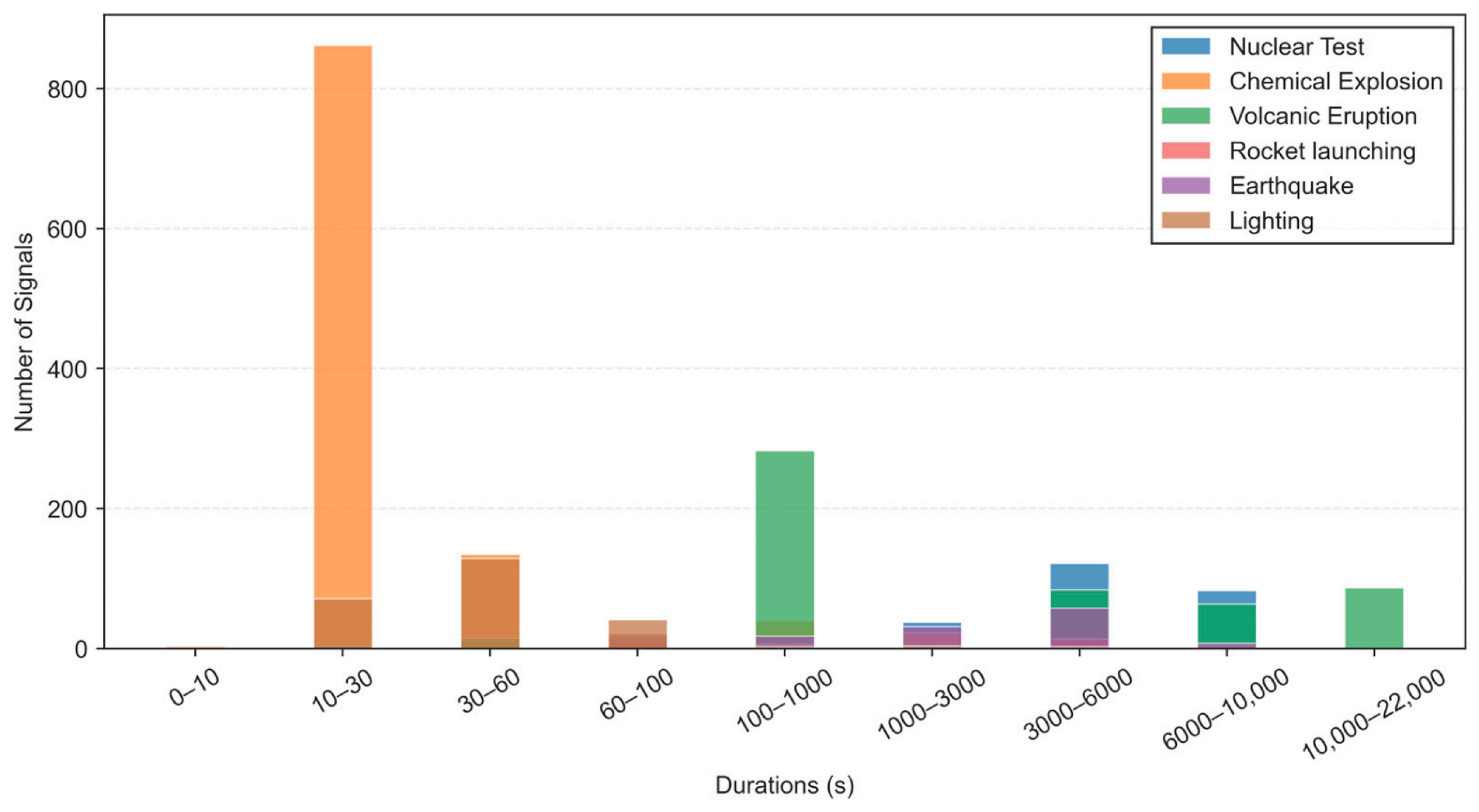
Duration distribution of signals from different infrasound event classes.

**Figure 3:**
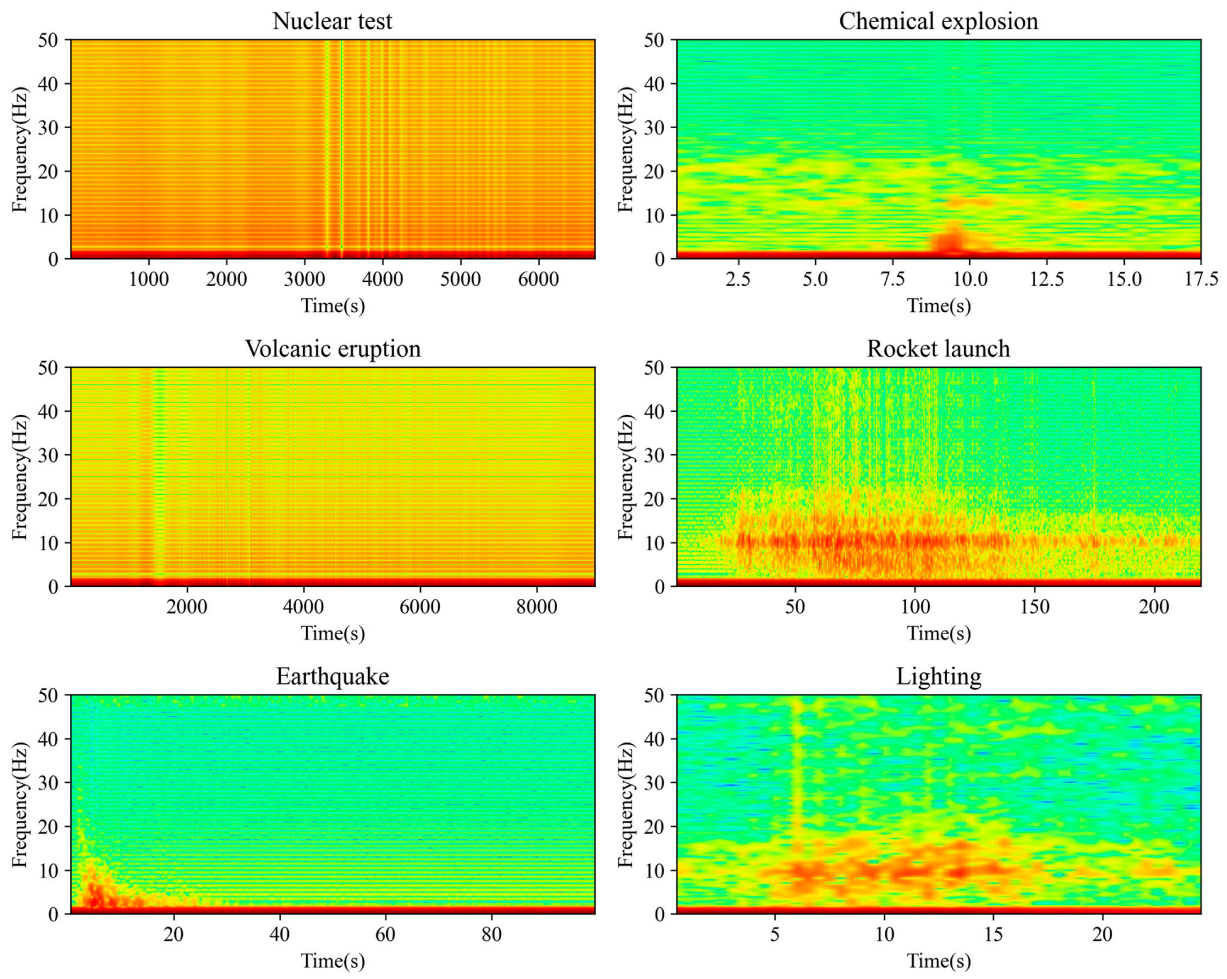
STFT time–frequency spectrograms of six types of signals show in Figure 1.

In the specific implementation, a uniform STFT processing pipeline is applied to all infrasound signals to generate spectrograms: the window function is a Hann window, the window length is fixed at 1 s, the overlap is set to 50%, the FFT length is 1024 points, and the frequency range covers 0 Hz to 50 Hz. To compress the dynamic range and enhance the distinguishability of weaker spectral features, the power spectrum is converted to logarithmic power (dB scale). Subsequently, each spectrogram is independently normalized on a per-sample basis using min–max normalization, scaling the pixel values linearly to the interval [0, 1], without employing global normalization across the dataset, in order to avoid interference from global statistical bias on individual sample features. No color mapping or any extraneous graphical elements are introduced in the entire process. Because the raw infrasound recordings span durations from seconds to thousands of seconds across event classes (Figure 2), the STFT procedure produces spectrograms that share an identical frequency dimension but vary substantially in their time. Prior to being fed into the convolutional neural network, every spectrogram is uniformly rescaled to a fixed size of 256 × 256. No cropping, concatenation, or padding is applied to the original signals; the complete physical evolution process of each signal is fully preserved. As shown in Figure 2, the signal duration distributions of different event types span wide ranges and exhibit substantial overlap across a time span of thousands of seconds. For instance, nuclear tests, volcanic eruptions, and earthquakes fall within overlapping duration intervals, while certain chemical explosion signals of very short duration are indistinguishable from lightning events in terms of duration. These results demonstrate that event types cannot be effectively differentiated by signal duration alone, and duration itself does not constitute a reliable classification cue. It should be noted that the STFT parameter configuration adopted here was not chosen arbitrarily but determined through systematic comparative experiments on the time–frequency representation of infrasound signals. The effectiveness of this parameter set has been thoroughly validated in our previously published methodological study [53]. Figure 3 shows the STFT time–frequency spectrograms corresponding to Figure 1.

### 2.2. 4Conv3Fc Network

Widely used classical CNN architectures include LeNet-5, AlexNet, GoogLeNet, VGGNet, and ResNet. The total number of layers of these five types of neural networks is shown in Table 2. Given the specific characteristics of the infrasound signal classification task, LeNet-5 and AlexNet are selected as the baseline models in this study based on the following considerations. First, with regard to data scale, infrasound signal datasets are typically much smaller than large-scale image datasets such as ImageNet. LeNet-5 has a simple architecture that is less prone to overfitting on limited data and ensures stable and efficient training, while AlexNet, with its moderate scale, achieves better generalization on moderately sized infrasound data than deeper models such as VGGNet, GoogLeNet, and ResNet, thus effectively avoiding severe overfitting caused by insufficient data. Second, in terms of model complexity and computational cost, VGGNet, GoogLeNet, and ResNet have large depths and enormous numbers of parameters, resulting in excessively high computational overhead that limits their practical application in infrasound signal processing scenarios requiring resource efficiency or rapid response. In contrast, LeNet-5 and AlexNet have considerably lower computational requirements, thereby facilitating extensive experimentation, optimization, and real-world deployment. Moreover, considering compatibility with the Bayesian CNN, this study aims to construct Bayesian CNNs for uncertainty quantification. Selecting LeNet-5 and AlexNet—both with relatively clear architectures and a moderate number of layers—as the backbone networks offers clear advantages over the other three deep models in terms of implementation difficulty, computational overhead, and result interpretability. Finally, from the perspective of representativeness in model development, LeNet-5 represents an early, landmark CNN architecture, whereas AlexNet marks a pivotal turning point in the deep learning renaissance. Together, these two models effectively cover representative network architectural paradigms spanning from foundational to early deep models. In summary, by comprehensively considering the scale of infrasound data, practical constraints on computational resources, the representativeness of model development, and the feasibility of integration with the Bayesian framework, LeNet-5 and AlexNet represent the most appropriate baseline model choices for this study.

**Figure 4:**
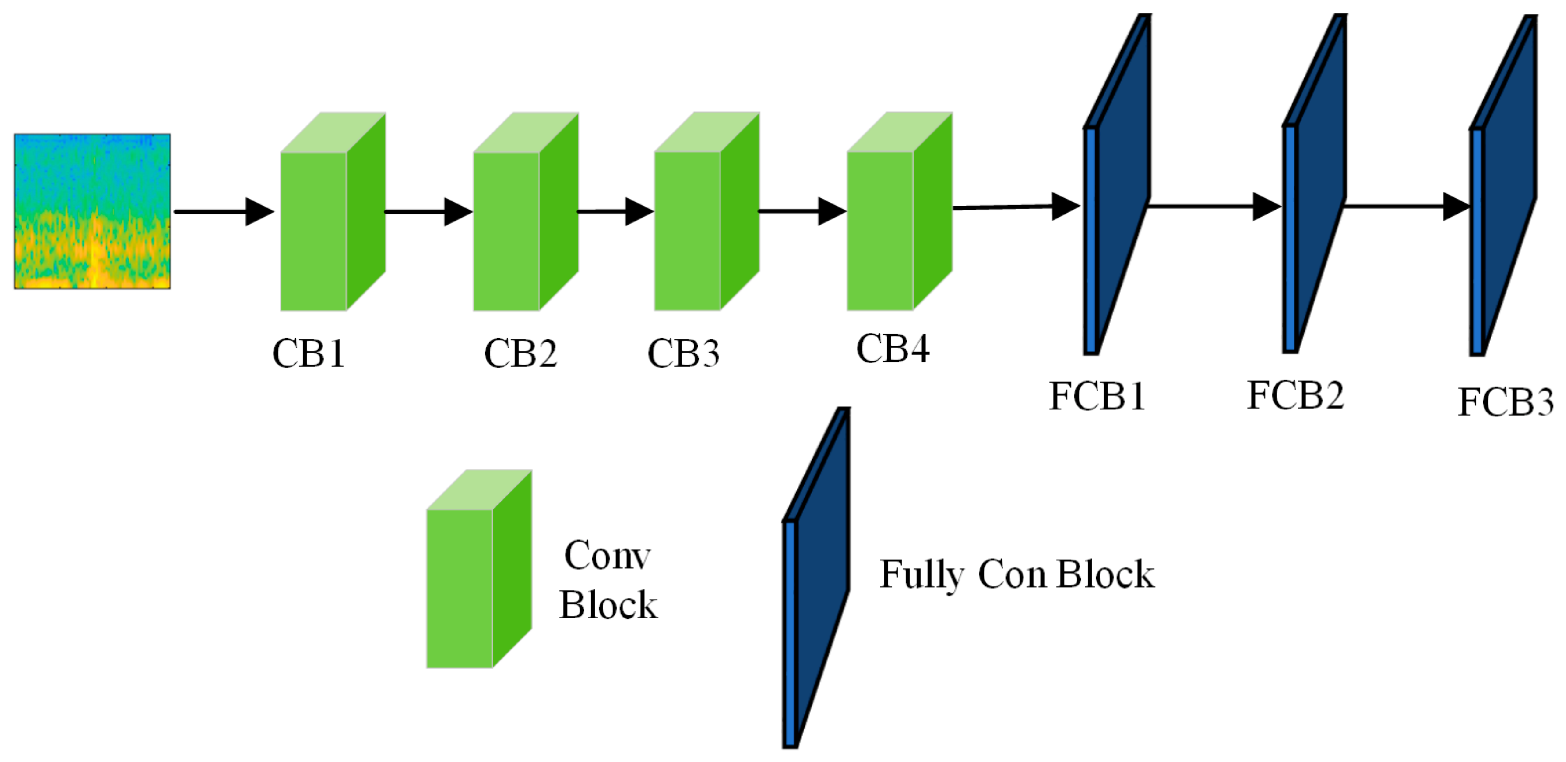
The structure of the 4Conv3Fc network.

This section constructs a 4Conv3Fc neural network architecture based on the idea of multi-scale convolution, and its structure is shown in Figure 4. By integrating a multi-scale feature extraction mechanism with time–frequency analysis methods, the network converts infrasound signals into time–frequency images for classification, aiming to achieve highly efficient performance. The architecture comprises four cascaded convolutional modules and three fully connected layers. The multi-scale convolutional mechanism employs a combination of 7 × 7, 5 × 5 and 3 × 3 convolutional kernels to enhance feature representation capabilities while diversifying the model’s receptive fields. Specifically, the kernel sizes follow a coarse-to-fine strategy tailored to the characteristics of infrasound time–frequency images: the initial 7 × 7 kernel captures global spectral structures and large-scale acoustic patterns; the 5 × 5 kernel then extracts intermediate-level features such as frequency-band interactions; the two subsequent 3 × 3 kernels further refine local time–frequency textures and transient details, while the consecutive stacking of small kernels effectively expands the receptive field without a substantial increase in parameter count. The number of convolutional layers (four) is chosen to provide sufficient hierarchical feature abstraction while mitigating the risk of overfitting given the limited size of infrasound datasets; deeper architectures were empirically found to degrade generalization performance. The three fully connected layers are adopted to offer adequate nonlinear mapping from the high-level features to the output classes, achieving a favorable balance between model capacity and computational efficiency.

**Figure 5:**
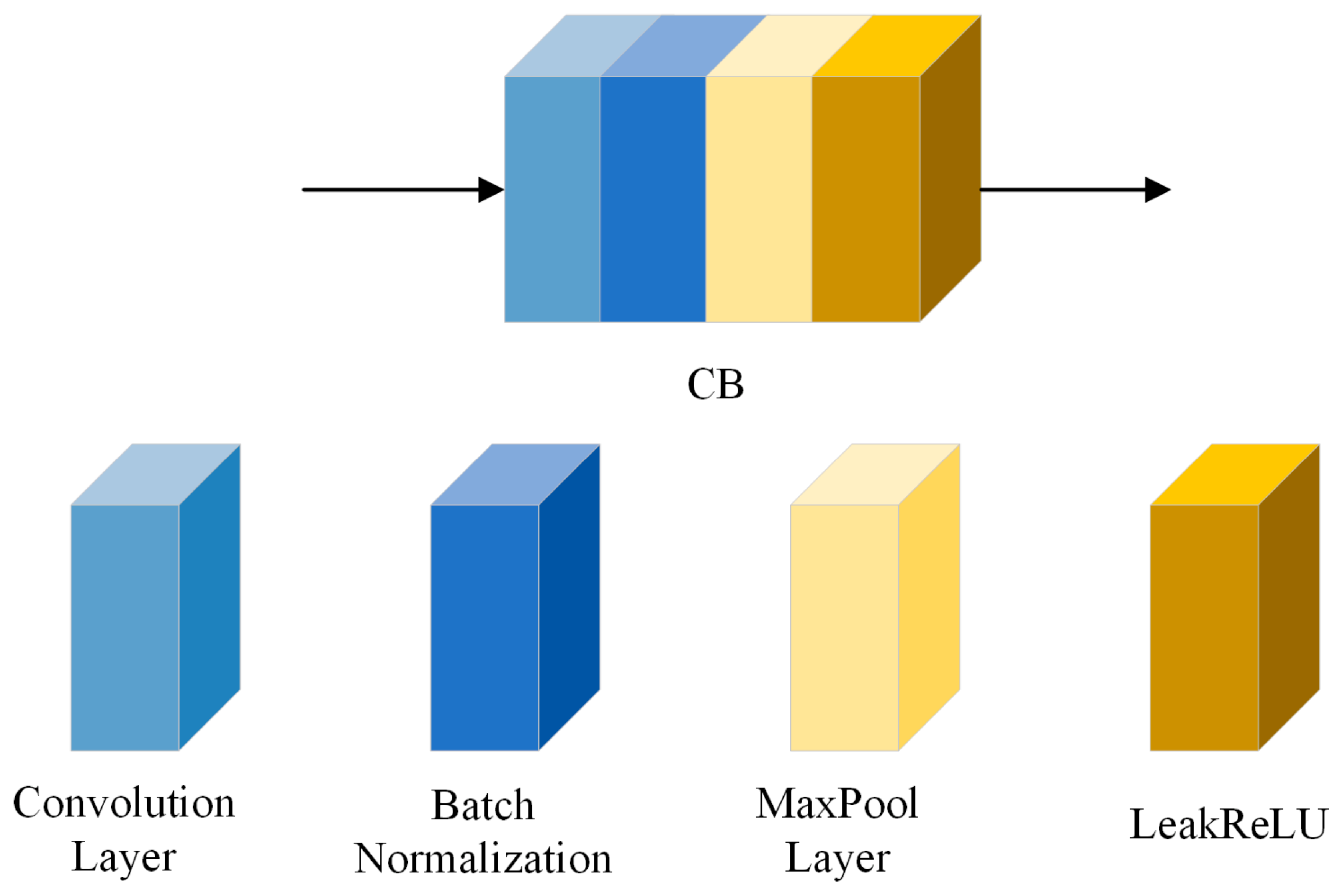
Structure of the convolutional block.

As shown in Figure 5, each convolutional module comprises four standardized components: a convolutional layer, a batch normalization (BN) layer, a max-pooling layer, and a nonlinear rectified activation unit. In the shallow layers of the network, a convolutional layer with a kernel size of 7 × 7 is employed to capture global feature information. As the network deepens, the convolutional kernel size is progressively reduced to 5 × 5 and 3 × 3, enabling fine-grained feature extraction from the time–frequency representation of infrasound signals. Compared with classical LeNet-5 and AlexNet, we have added BN after every convolutional layer to accelerate model convergence and performed feature-dimensionality reduction through a 2 × 2 max-pooling layer with a stride of 2. This design strategy, while preserving effective feature information, reduces the input dimensionality of subsequent network layers, thereby lowering the overall number of model parameters and improving the model’s generalization performance and computational efficiency. For nonlinear activation, this paper uses LeakyReLU instead of the traditional sigmoid and ReLU activation functions. LeakyReLU retains the advantages of ReLU—computational simplicity, mitigated gradient vanishing, and accelerated training—but its non-zero slope in the negative interval also prevents information loss caused by feature space compression.

### 2.3. Bayesian Convolutional Neural Network

To address the class imbalance problem in infrasound signal classification tasks, this study proposes a Bayesian-learning-based framework that transforms deterministic neural networks into a probabilistic inference paradigm by introducing probabilistic modeling into baseline models (LeNet-5, AlexNet, and 4Conv3Fc). Bayesian CNN, grounded in the Bayes’ theorem, treat deterministic network parameters as probability distributions. This approach transforms the learning process into a posterior inference problem, which can effectively quantify uncertainty and considerably enhance the interpretability and reliability of the model’s classification results [54,55,56].

#### 2.3.1. Bayes’ Theorem

Given a training dataset $D=\left(X,Y\right)$, where X represents time–frequency representation feature, Y is a class label, and the model parameters are **θ**, Bayes’ theorem can be expressed as

(1) $$p\left(\theta |D\right)=\frac{p\left(D,\theta \right)}{p\left(D\right)}=\frac{p\left(D|\theta \right)p\left(\theta \right)}{p\left(D\right)},$$

where $p\left(\theta |D\right)$ is the posterior probability, representing the probability distribution of model parameters θ given the observed data D. $p\left(\theta \right)$ is the prior probability, which represents the prior information of domain knowledge. $p\left(D|\theta \right)$ is the likelihood function, describing the probability of observing data D under parameters θ. And $p\left(D\right)$ is called the marginal likelihood or evidence, denoting the overall probability distribution of the observed dataset D.

In the context of supervised learning, the relationship between input X and label Y is critical. Specifically, the dataset D can be decomposed through the conditional probability $p\left(Y|X\right)$. Thus, it can be reformulated as

(2) $$p\left(\theta |X,Y\right)=\frac{p\left(Y|X,\theta \right)p\left(\theta \right)}{p\left(Y|X\right)},$$

where the marginal likelihood $p\left(Y|X\right)$ is expressed by the law of total probability,

(3) $$p\left(Y|X\right)=\int p\left(Y,\theta |X\right)d\theta =\int p\left(Y|\theta ,X\right)p\left(\theta |X\right)d\theta ,$$

Consequently, Bayes’ theorem can be further defined as

(4) $$p\left(\theta |X,Y\right)=\frac{p\left(y|X,\theta \right)p\left(\theta \right)}{\int p\left(y|\theta ,X\right)p\left(\theta |X\right)d\theta },$$

#### 2.3.2. Variational Inference

According to Section 2.3.1, solving the posterior distribution involves integral calculations in a high-dimensional parameter space, which is also a challenge in solving Bayesian CNN models. To address this issue, we use approximation methods to infer the true posterior distribution $p\left(\theta |D\right)$. Currently, the two most widely used approximation approaches are Markov Chain Monte Carlo (MCMC) and variational inference (VI). MCMC is a method that randomly samples from a probability distribution to estimate the true posterior distribution [57]. Its foundation is the Markov chain. In other words, the principle of this method is to construct a Markov chain whose stationary distribution is the target posterior [58] and then generate approximate samples through sampling techniques such as Metropolis–Hastings [59], Gibbs sampling [60], and Hamiltonian Monte Carlo (HMC) [61]. While MCMC achieves asymptotically exact sampling, its high computational cost and difficulty in assessing convergence [62] limit its applicability to deep CNNs. VI, as an alternative to MCMC, approximates the Bayesian posterior distribution. This method transforms the inference problem into a parameter optimization problem [63,64] by minimizing the Kullback–Leibler (KL) divergence between the variational distribution and the true posterior distribution. Compared with MCMC, VI sacrifices some accuracy to improve the computational efficiency of the model. Comparing the advantages and disadvantages of MCMC and VI, we choose VI to solve the model.

Suppose there is a variational distribution $q\left(\theta |\phi \right)$ parameterized by parameters ϕ. The goal of variational inference is to iteratively adjust the parameters ϕ such that the variational distribution $q\left(\theta |\phi \right)$ becomes as close as possible to the true posterior distribution $p\left(\theta |X,Y\right)$. We use the KL divergence to measure the difference between the variational distribution and the posterior distribution,

(5) $$KL\left(q\left(\theta |\phi \right)∥p\left(\theta |X,Y\right)\right)=\int q\left(\theta |\phi \right)\log\frac{q\left(\theta |\phi \right)}{p\left(\theta |X,Y\right)}d\theta ,$$

According to the above formula, calculating the KL divergence still requires computing the posterior distribution, and the difficulty remains. Decompose it further into

(6) $$KL\left(q\left(\theta |\phi \right)∥p\left(\theta |X,Y\right)\right)=\int q\left(\theta |\phi \right)\log\frac{q\left(\theta |\phi \right)}{p\left(\theta \right)}d\theta -\;\int q\left(\theta |\phi \right)\log p\left(Y|X,\theta \right)d\theta +\log p\left(Y|X\right),$$

The last term $\log p\left(Y|X\right)$ is not dependent on the parameter ϕ, and thus can be ignored. The optimization problem is equivalent to maximizing the evidence lower bound (ELBO) [65],

(7) $$ELBO=E_{q\left(\theta |\phi \right)}\left[\log p\left(Y|X,\theta \right)\right]-KL\left(q\left(\theta |\phi \right)∥p\left(\theta \right)\right),$$

The above process transforms the problem of minimizing KL divergence into the problem of maximizing the ELBO (or minimizing the variational free energy) [66]. For simplicity, we denote the variational free energy as

(8) $$F\left(D,\phi \right)=KL\left(q\left(\theta |\phi \right)∥p\left(\theta \right)\right)-E_{q\left(\theta |\phi \right)}\left[\log p\left(Y|X,\theta \right)\right],$$

where the former $KL\left(q\left(\theta |\phi \right)∥p\left(\theta \right)\right)$ depends on the prior distribution $p\left(\theta \right)$ and is called the complexity cost; the latter $E_{q\left(\theta |\phi \right)}\left[\log p\left(Y|X,\theta \right)\right]$ depends on $p\left(Y|X,\theta \right)$, which is called the likelihood cost.

The likelihood cost cannot be computed directly due to an integral. Monte Carlo sampling is used to approximate it:

(9) $$E_{q_{\phi }\left(\theta \right)}\left[\log p\left(Y|X,\theta \right)\right]=\int q_{\phi }\left(\theta \right)\log p\left(Y|X,\theta \right)d\theta \approx \frac{1}{K}\sum_{k=1}^{K}\log p\left(Y|X,\theta_{k}\right),$$

where K is the number of samplings of θ in $q_{\phi }\left(\theta \right)∼N\left(\theta ;\mu ,\sigma \right)$ during each training session.

Since the KL divergence term $KL\left(q_{\phi }\left(\theta \right)∥p\left(\theta \right)\right)$ is also intractable to compute exactly, we follow a stochastic variational method [67]. Therefore, our objective function can be further simplified to

(10) $$F\left(D,\phi \right)\approx \sum_{i=1}^{n}\log q_{\phi }\left(\theta^{\left(i\right)}|D\right)-\log p\left(\theta^{\left(i\right)}\right)-\log p\left(D|\theta^{\left(i\right)}\right),$$

The above formula consists of three terms. The first term $\log q_{\phi }\left(\theta^{\left(i\right)}|D\right)$ represents the variational posterior, which is a Gaussian distribution with mean μ and standard deviation σ,

(11) $$\log q_{\phi }\left(\theta^{\left(i\right)}|D\right)=\sum_{j}\log N\left(\theta_{j}|\mu ,\sigma \right),$$

The second term $\log p\left(\theta^{\left(i\right)}\right)$ corresponds to the log prior, defined as a zero-mean Gaussian distribution,

(12) $$\log p\left(\theta^{\left(i\right)}\right)=\sum_{j}\log N\left(\theta_{j}|0,\sigma_{p}^{2}\right),$$

The third term $\log p\left(D|\theta^{\left(i\right)}\right)$ is the likelihood function, which is the output of the network.

#### 2.3.3. Local Reparameterization Trick

Bayesian learning requires placing distributions over the model weights and then sampling from these distributions to obtain actual weights. As mentioned earlier, in this paper we use Gaussian distributions. To address the computational efficiency and gradient stability issues arising from weight sampling in Bayesian CNN, this study employs the reparameterization trick [68,69] in convolutional layers. This results in the subsequent equation for convolutional layer activation b,

(13) $$b_{j}=A_{i}*\mu_{i}+\epsilon_{j}⨀\sqrt{A_{i}^{2}*\left(\alpha_{i}⨀\mu_{i}^{2}\right)},$$

where $\epsilon_{j}∼N\left(0,1\right)$, $A_{i}$ denotes the receptive field, ∗ represents the convolution operation, and ⨀ indicates element-wise multiplication. The output of b serves as the posterior mean and is updated via the Adam optimizer. Additionally, the variance of the distribution is learned as a function of the mean. Shridhar et al. introduced a Softplus activation function to satisfy these two requirements [69]:

(14) $$Softplus\left(x\right)=\frac{1}{\beta }\log\left(1+e^{\beta x}\right),$$

In this study, we set β=1.

#### 2.3.4. Uncertainty Quantification

In classification tasks, we are interested in the predictive distribution $p_{D}\left(y^{*}|x^{*}\right)$, where $x^{*}$ is an unknown data sample and $y^{*}$ is the predicted class label.

(15) $$p_{D}\left(y^{*}|x^{*}\right)=\int p_{\theta }\left(y^{*}|x^{*}\right)p_{D}\left(\theta \right)d\theta ,$$

According to the objective function, the predicted distribution can achieve the expected unbiased estimator through T samplings,

(16) $$E_{q}\left[p_{D}\left(y*|x*\right)\right]=\int q_{\phi }\left(\theta |D\right)p_{\theta }\left(y|X\right)d\theta \approx \frac{1}{T}\sum_{t=1}^{T}p_{\theta_{t}}\left(y^{*}|x^{*}\right),$$

One of the primary advantages of integrating Bayesian learning into CNN is the ability to express aleatoric uncertainty and epistemic uncertainty. Aleatoric uncertainty usually arises from inherent noise in the data. This type of uncertainty cannot be reduced, even with the collection of additional data. Epistemic uncertainty refers to uncertainty inherent in the model itself, stemming from insufficient knowledge about the data. This uncertainty can be reduced by observing more data. Following the estimator in the above formula, we evaluate the uncertainty of our predictions through the variance,

(17) $${Var}_{q}\left(p\left(y^{*}|x^{*}\right)\right)=\underset{aleatoric}{\underbrace{\frac{1}{T}\sum_{t=1}^{T}diag\left({\hat{p}}_{t}\right)-{\hat{p}}_{t}{\hat{p}}_{t}^{T}}}+\underset{epistemic}{\underbrace{\frac{1}{T}\sum_{t=1}^{T}\left({\hat{p}}_{t}-\bar{p}\right){\left({\hat{p}}_{t}-\bar{p}\right)}^{T}}},$$

where $\bar{p}=\frac{1}{T}\sum_{t=1}^{T}{\hat{p}}_{t}$, representing the mean values of T samples ${\hat{p}}_{t}$, ${\hat{p}}_{t}=Softmax\left[f_{\theta_{t}}\left(x^{*}\right)\right]$, denotes the output of the network.

## 3. Results and Discussion

### 3.1. Experimental Setup

#### 3.1.1. Experimental Environment

We conducted extensive experiments and comparisons to evaluate the proposed method. The operating system of the computer used in this paper is Windows 10, the CPU is Intel(R) Core (TM) i9-14900K CPU @ 3.20 GHz, the graphics card is NVIDIA GeForce RTX 4090 D and 128 GB of RAM. The experiments are conducted using Python 3.9.19 via Jupyter Notebook bundled with Anaconda 1.11.0. All classification models are developed on the PyTorch 1.12.0 framework, and CUDA 11.6 is used to accelerate network training.

To prevent information leakage caused by splitting samples from the same physical event across different subsets, the dataset was partitioned into training, validation, and test sets using an event-level grouping strategy rather than random sample-level splitting. Specifically, all samples associated with the same physical event, identified by a unique event identifier, were assigned exclusively to the same subset. For the final performance evaluation, the event groups were randomly divided into training (80%), validation (10%), and test (10%) sets (Table 3). This ensures that no individual event is distributed across multiple sets. Although a single station or array may record multiple events, partitioning by events naturally allows data from the same station to appear in different sets while strictly preventing any event from being split. Moreover, because the test events were collected from different time periods, the atmospheric propagation conditions during testing differ markedly from those during training. This discrepancy increases the difficulty of the classification task and, consequently, enhances the credibility and generalizability of the results.

For the Bayesian models, all convolutional and fully connected layers were implemented with the local reparameterization trick, while batch normalization layers remained deterministic as they contain no trainable weights. The prior over weights was set to a zero-mean Gaussian with variance $\sigma_{p}^{2}=0.04$. The posterior means were initialized by drawing from $N\left(0,0.2^{2}\right)$, and the posterior variance parameters were initialized from $N\left(-5,0.2^{2}\right)$ and then passed through a softplus function to guarantee positivity. All models were trained for 200 epochs using the Adam optimizer with a learning rate of 0.001, a batch size of 32, and the evidence lower bound (ELBO) as the training objective. Regarding the weighting of the KL divergence term, we adopted the weight uncertainty method proposed by Blundell et al. [67]. The KL term was weighted by 1/N (with N being the number of training samples) to balance the likelihood and the complexity penalty, and the KL weight was linearly annealed from zero to its target value over the initial epochs to allow the model to first fit the data before regularizing toward the prior. During inference, T=50 Monte Carlo samples were drawn from the posterior to obtain stable estimates of the predictive distribution.

#### 3.1.2. Evaluation Metrics

In classification tasks, selecting appropriate evaluation metrics is crucial for identifying the best-performing classification model. Generally, evaluation metrics serve as tools to quantify the efficiency and performance of a classification algorithm. This paper evaluates our proposed infrasound signal classification model using multiple metrics, including accuracy, precision, recall, F1-score, and Cohen’s Kappa. These metrics are mathematically defined as follows:

(18) $$Accuracy=\frac{TP+TN}{TP+FP+TN+FN},$$

(19) $$Precision=\frac{TP}{TP+FP},$$

(20) $$Recall=\frac{TP}{TP+FN},$$

(21) $$F1-Score=2\times \frac{Precision\times Recall}{Precision+Recall},$$

(22) $$Cohen’s\;Kappa=\frac{P_{o}-P_{e}}{1-P_{e}},$$

where TP represents the true positive class, meaning that the true class is positive and the model correctly identifies it as positive. TN represents the true negative class, that is, the true class of the sample is negative and the model correctly identifies it as negative. FP represents the false positive class, meaning the true class is negative but the model incorrectly identifies it as positive. FN denotes the false negative, meaning the true class is positive but the model incorrectly identifies it as negative.

It should be particularly noted that Cohen’s Kappa is an important statistical indicator used to measure the consistency and reliability of a classifier. Unlike simple accuracy, this coefficient considers the impact of accidental consistency and provides a more accurate measure of model performance. $P_{o}$ is the observed proportional agreement, representing the actual proportion of agreement between evaluators across all samples. $P_{e}$ is the expected random agreement proportion, calculated assuming evaluators classify independently and randomly.

In addition to the five metrics mentioned above, this study employs a receiver operating characteristic (ROC) curve and precision–recall (PR) curve to validate the model’s performance. The ROC curve is derived from the confusion matrix and contrasts the true positive rate (TPR) against the false positive rate (FPR), where

(23) $$TPR=\frac{TP}{TP+FN},$$

(24) $$FPR=\frac{FP}{TN+FP},$$

A higher TPR coupled with a lower FPR (i.e., a steeper ROC) indicates better overall model performance. The larger the area under the precision–recall curve (PR-AUC), the better the balance between the model’s precision and recall.

In this study, all evaluation metrics—including accuracy, precision, recall, F1-score, and PR-AUC—are computed using macro-averaging across all classes. Macro-averaging calculates the metric independently for each class and then takes the unweighted mean, thereby treating all classes equally regardless of their sample sizes. This approach is deliberately chosen to ensure that the performance evaluation is not dominated by majority classes, providing an unbiased and rigorous assessment of model capability in highly class-imbalanced scenarios.

### 3.2. Analysis of Experimental Results

#### 3.2.1. Overall Classification Performance

To validate the effectiveness and superiority of the Bayesian CNN, we compared the classification performance of three baseline models—LeNet-5, AlexNet, and 4Conv3Fc—with their Bayesian versions. Table 4 shows the comparative results of the baseline models and their Bayesian versions on five core evaluation metrics (accuracy, precision, recall, F1-score, and Cohen’s Kappa coefficient). Without using any data augmentation techniques to increase sample size or enhance the training process, all baseline models achieved an average recognition accuracy exceeding 92%. Critically, minority classes also exhibited strong performance: for instance, the per-class F1-scores for earthquake and lightning events remained above 0.88 across all architectures, demonstrating that the high average accuracy is not solely driven by the dominant chemical explosion class. The accuracies of the three Bayesian CNN models were 97.26%, 98.83%, and 99.14%, which were 2.69, 6.58, and 2.68 percentage points higher than the baseline models, respectively. Notably, the AlexNet architecture exhibited the largest absolute accuracy gain. Its Bayesian counterpart, however, retained comparatively high variability on the smallest class (rocket launch, PR-AUC 0.927 ± 0.086), indicating that Bayesian inference mitigates but does not fully eliminate the instability introduced by the architecture’s high capacity. Compared with the baseline models, the Bayesian CNN improved precision by 2.71, 6.91, and 2.72 percentage points and recall by 2.96, 7.12, and 2.83 percentage points. In our experiments, the dataset showed marked imbalance; therefore, we focused on the F1-score. The experimental results showed that the F1-score increased by 2.94, 7.2, and 2.83 percentage points, indicating that our method can effectively coordinate the balance of precision and recall. Furthermore, we found that the Cohen’s Kappa coefficient increased by 4.46, 8.86, and 4.00 percentage points. The improvement in Cohen’s Kappa indicates that the model’s discriminative ability is considerably better than random guessing; combined with an event-level splitting strategy, this eliminates the confounding effect of data leakage on the performance gains, thereby confirming that the improvement arises from enhanced discriminative capability.

Compared with deterministic baseline models, the Bayesian CNN demonstrates improvements in classification accuracy, precision, recall, and F1-score. We hypothesize that this improvement can be attributed to the Bayesian framework’s ability to model uncertainty and its inherent ensemble effect, both of which may contribute to better generalization. A detailed isolation of these contributing factors remains an interesting avenue for future work. Deterministic models typically rely on point estimates of weights, which are prone to overfitting, especially when data is limited or noisy. In contrast, the Bayesian approach introduces prior distributions and posterior inference, treating weights as probability distributions. During prediction, multiple possible weight configurations are sampled to average the results, analogous to an implicit model ensemble. This effectively reduces variance and improves adaptability to unseen data. Furthermore, the Bayesian framework inherently provides a regularization effect by constraining the parameter space through prior knowledge, preventing the model from over-relying on noise or outliers in the training data. Simultaneously, uncertainty estimation enables the model to more accurately assess prediction confidence, thereby better balancing precision and recall in classification decisions and ultimately improving comprehensive metrics such as the F1-score. Therefore, adopting the Bayesian framework not only enhances model calibration and stability through uncertainty modeling but also optimizes classification performance via ensemble effects and regularization mechanisms, leading to superior performance in complex tasks. The separability among different infrasound signal classes fundamentally arises from the distinct imprints left on spectrograms by their physical source mechanisms and propagation paths. Nuclear tests, typically underground or near-surface explosions, produce infrasound signals rich in low-frequency components, with wave train durations extending to hundreds of seconds and clear dispersive structures formed by stable propagation paths. Chemical explosions, mostly surface or air bursts, exhibit relatively broad spectra, shorter durations, and highly variable waveforms influenced by burst height and yield. Volcanic eruptions are often accompanied by sustained tremor or explosion sequences, and their spectrograms display stable or time-varying narrowband harmonic energy that can persist for thousands of seconds. The acoustic signals of rocket launches comprise multiple stages such as engine ignition and transonic booms, with spectra characterized by markedly non-stationary evolution. Earthquake-induced infrasound signals are generally generated through ground–atmosphere coupling, with energy concentrated in lower frequency bands and relatively simple waveforms. Thunder from lightning is impulsive and broadband, exhibiting dispersed spectral energy. The model achieves high recall and high precision across all classes, particularly demonstrating a strong detection capability for the sparsely sampled rocket launch and nuclear test classes. This indicates that the network has learned these physically distinguishable acoustic features from the spectrograms rather than relying solely on superficial statistical artifacts.

Additionally, as shown in Table 4, the Bayes 4Conv3Fc model proposed in Section 2.2 exhibits the best overall performance in our infrasound signal dataset. Its average accuracy reaches 99.14%, indicating stronger overall classification capability. The precision is 99.09%, which means that the great majority of positive predictions are correct, corresponding to a very low false-positive rate. The recall of 99.05% reflects minimal missed detections, confirming its effectiveness in identifying true positive samples. The model achieves an outstanding balance between precision and recall, as evidenced by its F1-score of 99.05%. Furthermore, the Cohen’s Kappa coefficient of 98.98% underscores the model’s high reliability in real-world applications, validating its practical utility for imbalanced infrasound signal classification tasks.

**Figure 6:**
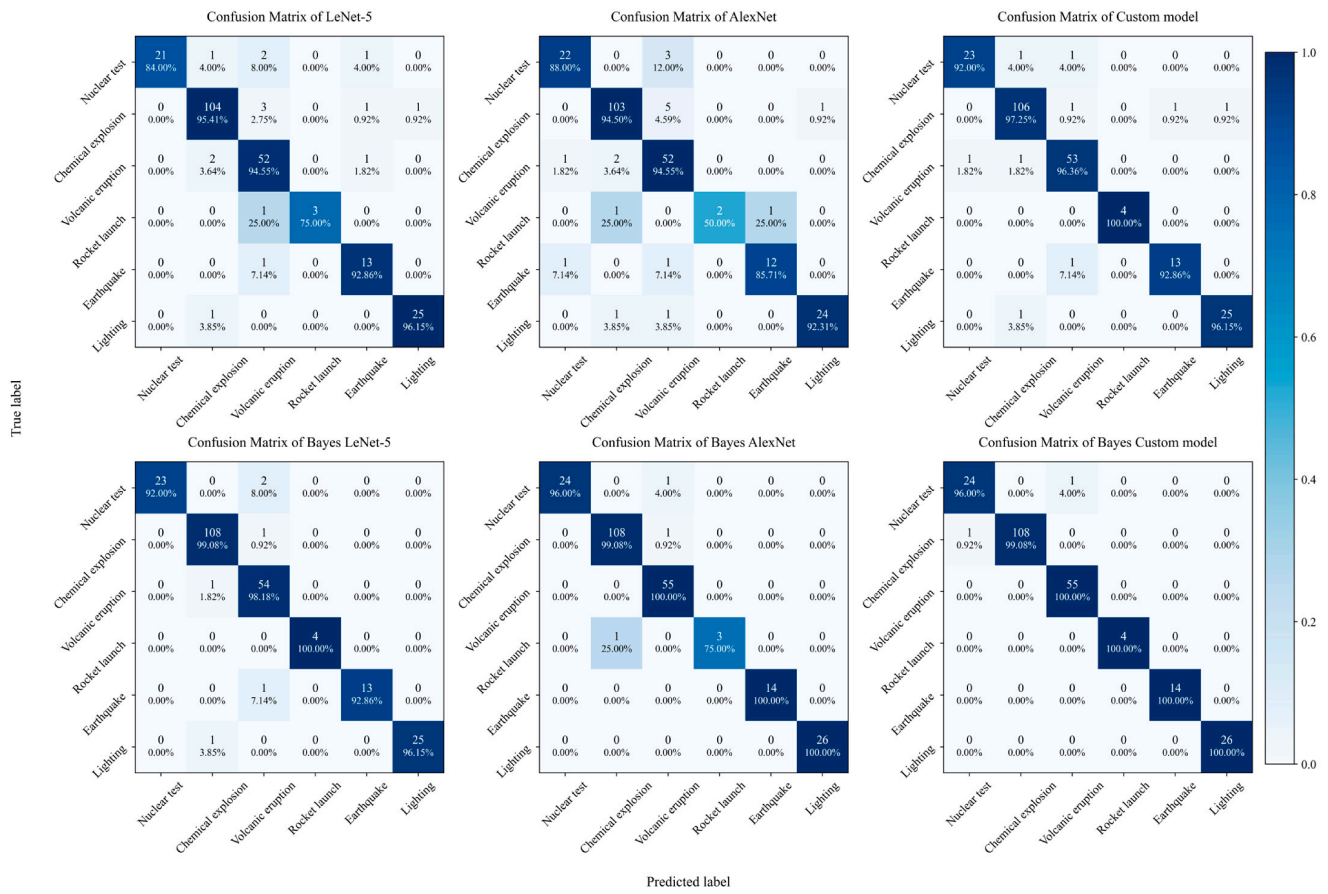
Confusion matrices of different models.

This study substantially improved the classification performance of the model for the class imbalance problem in the infrasound signal classification task through a Bayesian CNN. The confusion matrix visualized in Figure 6 demonstrates the excellent classification capabilities of the custom-designed 4Conv3Fc model. Chemical explosions, constituting nearly half of the dataset, were classified with 99.08% accuracy with the Bayes 4Conv3Fc model, marking a 1.83 percentage points improvement over the baseline model. Although the numbers of samples for nuclear tests (25), rocket launches (4), earthquakes (14), and lightning events (26) were small, the proposed model attained accuracies of 96%, 100%, 100%, and 100%, respectively. For the baseline LeNet-5 and its Bayes LeNet-5, the accuracies for the four signal classes of nuclear tests, chemical explosions, volcanic eruptions and rocket launches increased by 8, 3.67, 3.63, and 25 percentage points, with a maximum single-class improvement of 25 percentage points. For the baseline AlexNet and its Bayes AlexNet, the accuracies of all six classes were improved by 8, 4.58, 5.45, 25, 14.29, and 7.69 percentage points, respectively, with a maximum single-class improvement of 25%. For the baseline 4Conv3Fc model and its Bayes 4Conv3Fc, the recognition accuracies for the five signal classes of nuclear test, chemical explosion, volcanic eruption, earthquake, and lightning increased by 4, 1.83, 3.64, 7.14, and 3.85 percentage points, respectively, with a maximum single-class improvement of 7.14 percentage points. Notably, the custom 4Conv3Fc model exhibited the best performance among the baseline models, indicating that its architectural design is more suitable for infrasound classification. Its performance improved further under the Bayesian framework.

#### 3.2.2. ROC-AUC and PR-AUC Results

Given the highly class-imbalanced nature of the dataset, this study employs a dual-metric evaluation system comprising the area under the receiver operating characteristic curve (ROC-AUC) and the area under the precision–recall curve (PR-AUC). However, because ROC-AUC can be overly optimistic in imbalanced scenarios, our primary emphasis is placed on PR-AUC, which more faithfully reflects a model’s ability to correctly identify minority-class samples. ROC-AUC values are reported for completeness but are discussed only briefly.

**Figure 7:**
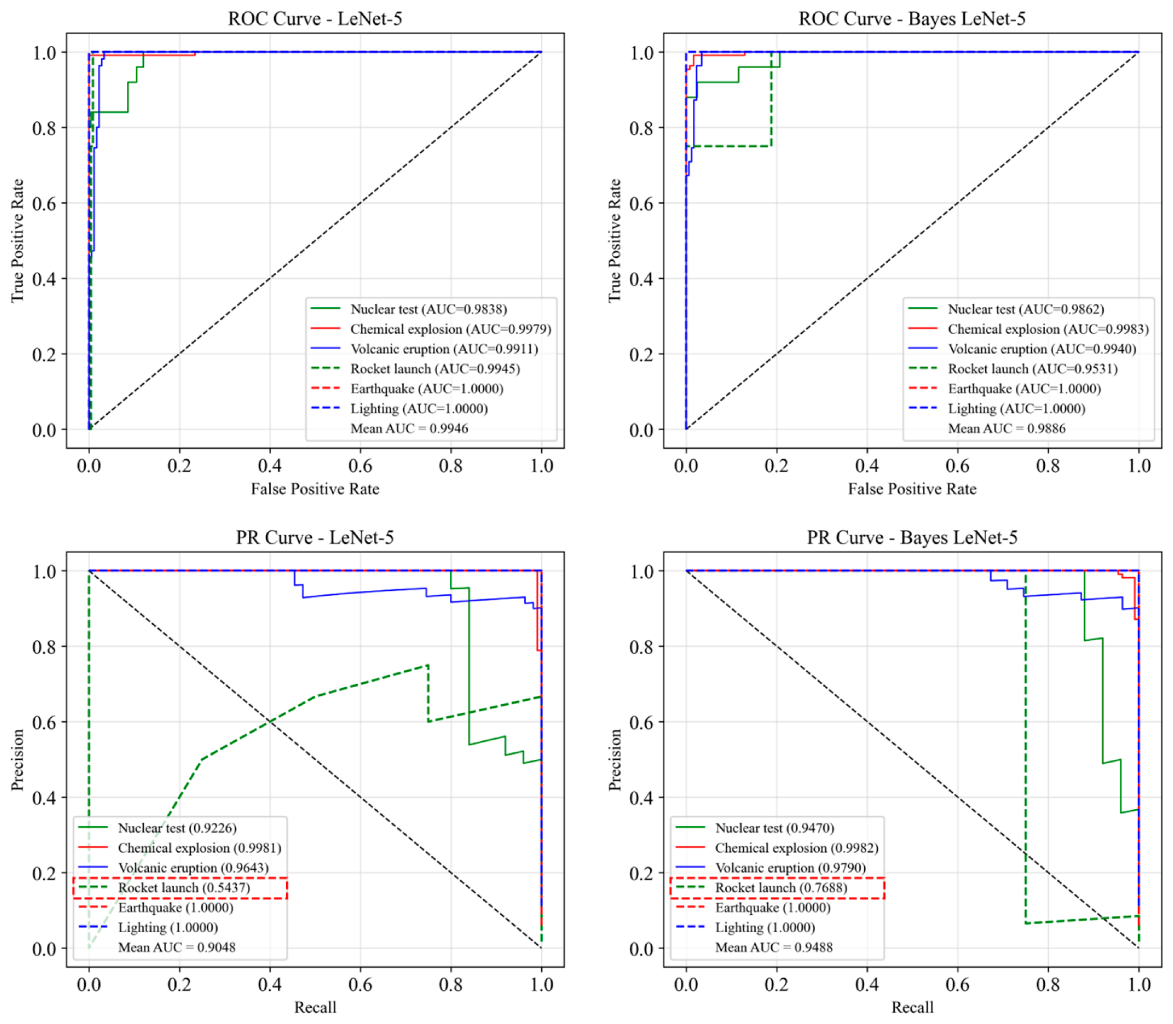
The ROC-AUC curves and PR-AUC curves of LeNet-5 and Bayes LeNet-5.

Figure 7 presents the PR-AUC and ROC-AUC curves for LeNet-5 and Bayes LeNet-5. With respect to PR-AUC, the Bayes LeNet-5 model achieved an average of 0.9488, slightly outperforming the baseline LeNet-5. Notably, for the rocket launch class—the most challenging minority category—the PR-AUC increased from 0.5437 to 0.7688, an absolute gain of 0.225, indicating that Bayesian learning substantially strengthens the model’s capability to extract discriminative features from limited samples. For the nuclear test class, the PR-AUC also rose from 0.9470 to a near-perfect level. For well-represented classes such as chemical explosions and volcanic eruptions, both models attained high PR-AUC values above 0.96, demonstrating effective feature capture.

**Figure 8:**
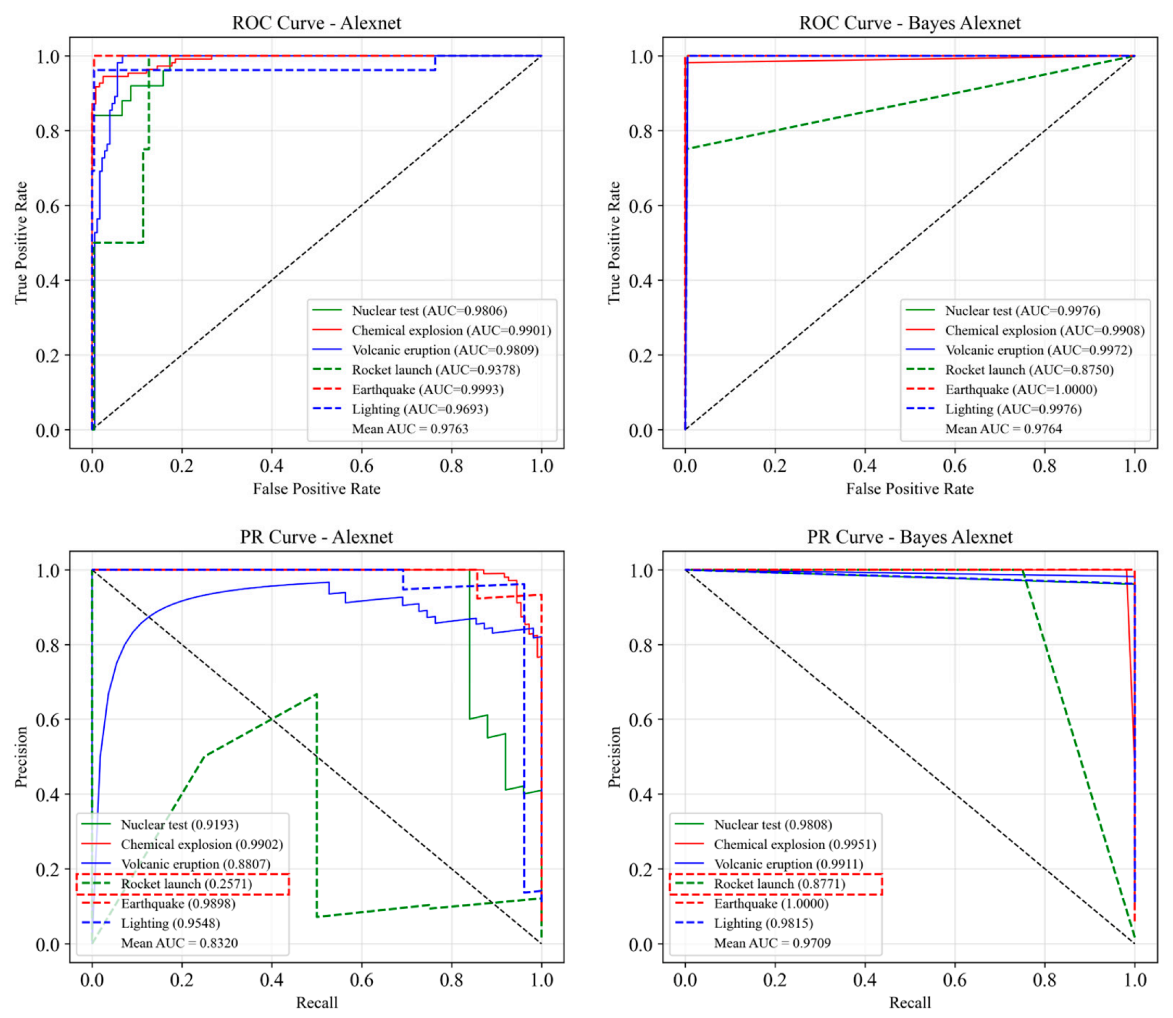
The ROC-AUC curves and PR-AUC curves of AlexNet and Bayes AlexNet.

Figure 8 illustrates the curves for AlexNet and Bayes AlexNet. The average PR-AUC of Bayes AlexNet reached 0.9709, a marked improvement over AlexNet (0.8320). The most striking gain was again observed for rocket launches, where the PR-AUC increased from 0.2571 to 0.8771, an absolute gain of 0.62, demonstrating that the Bayesian approach largely remedies the poor minority-class performance caused by AlexNet’s high model complexity and data scarcity. Consistent improvements in PR-AUC were also recorded for nuclear tests, lightning, and volcanic eruptions.

**Figure 9:**
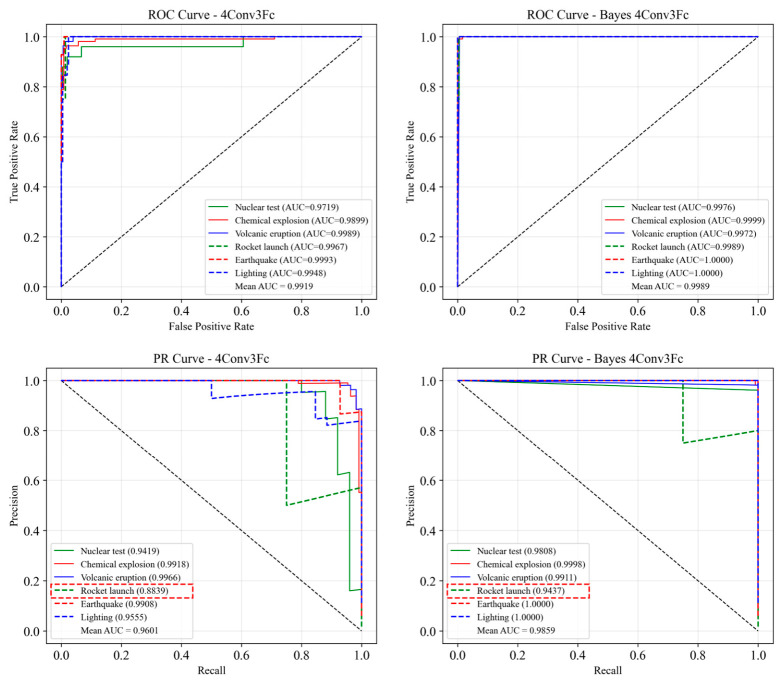
The ROC-AUC curves and PR-AUC curves of 4Conv3Fc and Bayes 4Conv3Fc.

Figure 9 shows the results for the 4Conv3Fc and Bayes 4Conv3Fc models. The Bayes 4Conv3Fc achieved an average PR-AUC of 0.9859, outperforming the 4Conv3Fc baseline (0.9701). For rocket launches, the PR-AUC increased from 0.8839 to 0.9437, an absolute gain of 0.0598, and for nuclear tests, it rose to 0.988. All other classes maintained PR-AUC values above 0.98, confirming the architecture’s robustness and the additional benefit of Bayesian learning.

Overall, across all three architectures, the Bayesian CNN variants consistently yielded higher PR-AUC compared with their deterministic counterparts, with average absolute improvements of 4.4, 13.89, and 2.58 percentage points, respectively. Although the corresponding ROC-AUC values remained high for most models (detailed in Figure 7, Figure 8 and Figure 9), the divergence between ROC-AUC and PR-AUC is most apparent for the rocket launch class under LeNet-5 and AlexNet: PR-AUC improved substantially while ROC-AUC sometimes decreased. This discrepancy arises precisely because ROC-AUC is insensitive to changes in the positive-to-negative sample ratios under severe imbalance, whereas PR-AUC reliably reflects the model’s true positive predictive capacity. Hence, PR-AUC is the more trustworthy metric for the present evaluation.

To systematically quantify model robustness, multiple repeated experiments were conducted. Table 5 summarizes the means and standard deviations of ROC-AUC, while Table 6 focuses on the PR-AUC statistics, which constitute the core evidence for model assessment.

From the PR-AUC results in Table 6, the Bayes 4Conv3Fc model consistently achieves the highest or near-highest values across all event categories, with remarkably low standard deviations, underscoring its superior stability and accuracy. It leads in chemical explosions (0.999 ± 0.003) and rocket launches (0.994 ± 0.019) and remains highly competitive for volcanic eruptions and nuclear tests. Bayes LeNet-5 outperforms its deterministic baseline in every category, most notably raising nuclear test PR-AUC from 0.944 to 0.984 and earthquake PR-AUC from 0.961 to 0.990, while also substantially reducing variance. The AlexNet baseline exhibits severe performance degradation for rocket launches (0.587 ± 0.197) with very high variability, confirming that complex models without Bayesian treatment are ill-suited for small and imbalanced data. Incorporating Bayesian inference (Bayes AlexNet) dramatically lifts the rocket launch PR-AUC to 0.927 and narrows the gap with other classes, although some variability remains. Overall, the Bayesian variants (Bayes LeNet-5 and Bayes 4Conv3Fc) yield not only higher mean PR-AUC but also lower standard deviations, demonstrating enhanced reliability. Based on these results, Bayes 4Conv3Fc is recommended as the preferred model for high-precision infrasound signal classification tasks such as nuclear test monitoring, while Bayes LeNet-5 offers a favourable accuracy–complexity balance given its markedly smaller parameter count, suitable for resource-constrained scenarios like earthquake and chemical explosion classification.

The substantial PR-AUC gains, particularly for the smallest class (rocket launch), confirm that Bayesian learning effectively addresses the challenges of imbalanced data. Compared with deterministic models, Bayesian CNN model weight uncertainty and perform approximate Bayesian model averaging during inference. This mechanism acts as a powerful regularizer that prevents overfitting to majority classes and creates an ensemble effect, enabling the model to capture the unique non-stationary time–frequency patterns of minority signals—such as the rapid spectral energy migration during a rocket launch. Consequently, the decision boundary is less biased toward dominant classes, leading to a superior precision–recall trade-off. The consistently high PR-AUC values for chemical explosions and volcanic eruptions further verify their robust physical separability in the time–frequency domain. Thus, by fundamentally altering the learning and inference paradigm, Bayesian CNN provide a more faithful and reliable classification framework for highly imbalanced infrasound datasets.

Compared with deterministic baseline models, Bayesian CNN demonstrate superior classification performance on imbalanced datasets. The fundamental reason lies in the Bayesian framework’s inherent ability to model uncertainty and its ensemble effect, which effectively mitigates the model’s overfitting to majority classes while enhancing its capability to recognize scarce minority-class samples. In imbalanced datasets, deterministic models learning through optimization algorithms (such as gradient descent) tend to strongly favor the numerically dominant classes, as following the majority path most directly reduces the overall loss function. However, this leads to insufficient learning of minority-class features and biases the decision boundary in favour of the majority classes. In contrast, the Bayesian CNN treats model parameters as probability distributions and learns through Bayesian inference. This mechanism offers several key advantages: First, the prior distribution on weights (e.g., the Gaussian prior) acts as a powerful regularizer, constraining parameter freedom and preventing the model from overconfidently memorizing noisy patterns of the majority classes. This encourages the learning of more generalizable features. Second, during prediction, the Bayesian CNN approximates Bayesian model averaging through multiple forward-pass sampling, which is equivalent to integrating a large ensemble of sub-models that share the same prior but exhibit slight variations. This ensemble effect ensures that predictions for sparse minority-class samples are not compromised by the bias of a single point estimate of weights. Some sub-models are likely to capture critical minority-class features, thereby improving recall through collective decision-making. Ultimately, this quantification of uncertainty yields more conservative confidence estimates. When faced with samples with ambiguous features, its predicted probabilities remain closer to uniform rather than saturating at extreme values, directly leading to a better precision–recall trade-off and reflected in a higher F1-score. Thus, rather than directly modifying the loss function to balance classes, Bayesian CNN fundamentally change the paradigm of model learning and inference, endowing the model with greater robustness and fairness from the ground up, enabling it to excel in imbalanced data environments.

#### 3.2.3. K-Fold Cross-Validation

In this section, we systematically evaluate the robustness and generalization capabilities of all models on the class-imbalanced infrasound datasets using 10-fold cross-validation. As discussed previously, if such event-level metadata are unavailable, generalization performance cannot be adequately assessed, because random sample-level splitting would allow highly correlated samples from the same event to appear in both the training and validation folds, yielding overly optimistic and unreliable evaluation results. Following this event-level splitting strategy, we obtain statistical results for seven core evaluation metrics, as shown in Table 7.

As shown in Table 7, for the classic baseline models (LeNet-5 and AlexNet), the transition from deterministic to Bayesian CNN reduced the standard deviations of accuracy, precision, and recall from about 1.4-1.5% to below 0.8% and the standard deviation of the F1-score from 1% to 0.8%. For our custom model (4Conv3Fc), the Bayesian variant decreased the standard deviations of these four metrics from 1% to 0.3%. These improvements demonstrate that Bayesian inference enhances model robustness and achieves a more stable balance between precision and recall. Although the deterministic AlexNet exhibited the lowest Cohen’s Kappa coefficient (0.896 ± 0.037), it still exceeded the lower bound of the “almost perfect agreement” threshold (0.81–1.0) proposed by Landis and Koch [70]. The Bayesian counterparts of the three models attained Cohen’s Kappa coefficients of 0.964 ± 0.023, 0.985 ± 0.013, and 0.990 ± 0.009, respectively, substantially surpassing the lower bound of “almost perfect agreement” and confirming a statistically marked association between predictions and true labels. The ROC-AUC values of all six models ranged from 0.984 to 0.999, approaching the theoretical optimum and indicating excellent overall discriminative capability. The deterministic baselines LeNet-5, AlexNet, and 4Conv3Fc achieved PR-AUC values of 0.958 ± 0.026, 0.895 ± 0.038, and 0.984 ± 0.011, respectively, revealing that AlexNet had the weakest ability to distinguish positive from negative classes, while 4Conv3Fc performed best—consistent with the findings in Section 3.2.1. By incorporating Bayesian inference, the average PR-AUC of all three architectures exceeded 0.98, with standard deviations below 0.02. This indicates that the Bayesian models sustain a favourable precision–recall trade-off for the minority classes across the full range of decision thresholds and validates the effectiveness of the proposed Bayesian CNN framework in real-world scenarios characterized by imbalanced data distributions.

To examine whether the performance improvement in Bayesian convolutional neural networks originates solely from class imbalance handling, we replaced the loss with cross-entropy weighted by the nverse training-set class frequencies while keeping the network architectures and training configurations identical. This yielded three deterministic weighted models—LeNet-5-weights, AlexNet-weights, and 4Conv3Fc-weights—which were systematically compared with the original deterministic models and their Bayesian counterparts. Table 8, Table 9 and Table 10 report the precision, recall, and F1-score for the six event classes.

Experimental results show that class weighting generally improves the recall and F1-score of deterministic models, although its effect on precision is not entirely consistent. Taking AlexNet as an example, class weighting raises the recall for the rocket launch class from 0.525 to 0.725 and the F1-score from 0.583 to 0.723; for the earthquake class, recall and F1-score increase from 0.821 and 0.850 to 0.886 and 0.891, respectively. Similar improvements are observed for LeNet-5 and 4Conv3Fc, confirming that class weighting can alleviate the classification bias caused by imbalanced class distributions.

Nevertheless, Bayesian CNNs consistently and markedly outperform the corresponding class-weighted deterministic models across all three architectures and nearly all event classes. The six-class average F1-scores of Bayes LeNet-5, Bayes AlexNet, and Bayes 4Conv3Fc are approximately 0.952, 0.974, and 0.983, while those of their class-weighted counterparts are 0.937, 0.886, and 0.951, respectively. The gap is particularly striking for the AlexNet architecture: Bayes AlexNet achieves F1-scores of 0.910, 0.993, and 0.982 for rocket launch, earthquake, and volcanic eruption, markedly surpassing the 0.723, 0.891, and 0.889 obtained by AlexNet-weights. For the earthquake class, weighting only lifts recall from 0.821 to 0.886, whereas the Bayesian model drives both recall and precision to 0.993, pushing the F1-score from 0.891 to 0.993. Even for the relatively well-represented chemical explosion class, Bayes AlexNet attains an F1-score of 0.992, exceeding the 0.957 of the weighted model.

Beyond these mean improvements, a class-level examination with standard deviations (Table 8, Table 9 and Table 10) reveals that Bayesian models also confer markedly enhanced stability across data splits—an aspect critical for operational monitoring. For the nuclear test class, Bayesian models consistently outperform their deterministic counterparts on all metrics. Bayes 4Conv3Fc achieves the highest F1-score of 0.978 ± 0.020, substantially surpassing deterministic 4Conv3Fc (0.932 ± 0.041), as well as Bayes LeNet-5 (0.950 ± 0.017) and Bayes AlexNet (0.974 ± 0.023). Deterministic AlexNet exhibits the poorest performance for this class (F1: 0.877 ± 0.036, precision: 0.901 ± 0.050), indicating that high model complexity without Bayesian regularization leads to overfitting on limited rare-event data. The Bayesian treatment elevates its F1-score to 0.974 ± 0.023 with substantially reduced standard deviation, underscoring the stabilizing effect of weight uncertainty modeling.

The performance disparity between deterministic and Bayesian models is most pronounced for the rocket launch class—the most severely underrepresented category with only 33 samples. Bayes 4Conv3Fc attains perfect precision and high recall (1.000 ± 0.000 and 0.950 ± 0.105), yielding an F1-score of 0.971 ± 0.060, which dramatically surpasses deterministic 4Conv3Fc (0.887 ± 0.154). Bayes AlexNet demonstrates a dramatic improvement over its deterministic counterpart, with F1-score rising from 0.583 ± 0.243 to 0.910 ± 0.111 and precision from 0.733 ± 0.306 to 1.000 ± 0.000. The very large F1-score standard deviation of deterministic AlexNet (0.243) reveals that this architecture is highly unstable when trained on extremely scarce data; the Bayesian framework effectively mitigates this instability. The near-perfect precision for rocket launches indicates that Bayesian models rarely produce false alarms when predicting this class—a highly desirable property given the substantial costs of false alarms in operational settings.

For the earthquake class (137 samples), Bayes AlexNet achieves near-perfect performance (F1: 0.993 ± 0.015), representing the most marked improvement over its deterministic counterpart (0.850 ± 0.063). This striking enhancement further illustrates the effectiveness of Bayesian inference in enabling complex architectures to learn robustly from limited data.

A critical observation across all minority classes is that Bayesian models systematically reduce standard deviations while improving mean performance. For rocket launches, the F1-score standard deviation decreases from 0.243 (deterministic AlexNet) to 0.111 (Bayes AlexNet); for nuclear tests, it drops from 0.041 (4Conv3Fc) to 0.020 (Bayes 4Conv3Fc). This consistent pattern confirms that the Bayesian framework not only improves classification accuracy but also substantially enhances model robustness across different data splits.

These results reveal that the advantages of Bayesian models cannot be fully explained by class re-weighting alone. Class-weighted cross-entropy merely rescales the error signals, whereas Bayesian learning introduces adaptive regularization through a prior over weight distributions and performs approximate Bayesian model averaging (i.e., implicit ensembling) via Monte Carlo sampling during prediction. These mechanisms synergistically suppress overfitting to majority classes and substantially enhance generalization for tail classes and hard samples. Consequently, beyond mitigating class imbalance, Bayesian posterior modeling of parameters improves decision robustness under conditions of limited samples, inter-class feature overlap, and observation noise, enabling a substantial increase in recall while maintaining high precision—especially in severely imbalanced scenarios.

In summary, class-weighted cross-entropy provides a strong deterministic imbalance baseline and validates the additional performance gains of Bayesian CNN over this baseline. The class-level analysis further demonstrates that Bayesian learning confers the greatest benefits to the most challenging categories: weight uncertainty modeling serves as an effective regularizer that prevents overfitting to the dominant majority classes while preserving sensitivity to the discriminative features of rare events. These properties make the proposed Bayesian CNN framework particularly well-suited for real-world applications such as nuclear test monitoring and rocket launch identification. Nevertheless, to strictly attribute the gains to Bayesian posterior marginalization itself, comparisons with explicit deep ensembles and MC Dropout remain necessary, which will be pursued in future work.

#### 3.2.4. Uncertainty Estimation and Calibration

To our knowledge, this work is the first to explore the use of Bayesian CNNsto quantify epistemic uncertainty and aleatoric uncertainty in infrasound signal classification models. Deterministic CNN do not provide a principled estimate of predictive uncertainty. Because Bayesian CNN provide posterior distributions, uncertainty can be estimated uncertainty according to Section 2.3.4. Thus, this section focuses only on the Bayesian CNN and compare their performance.

**Figure 10:**
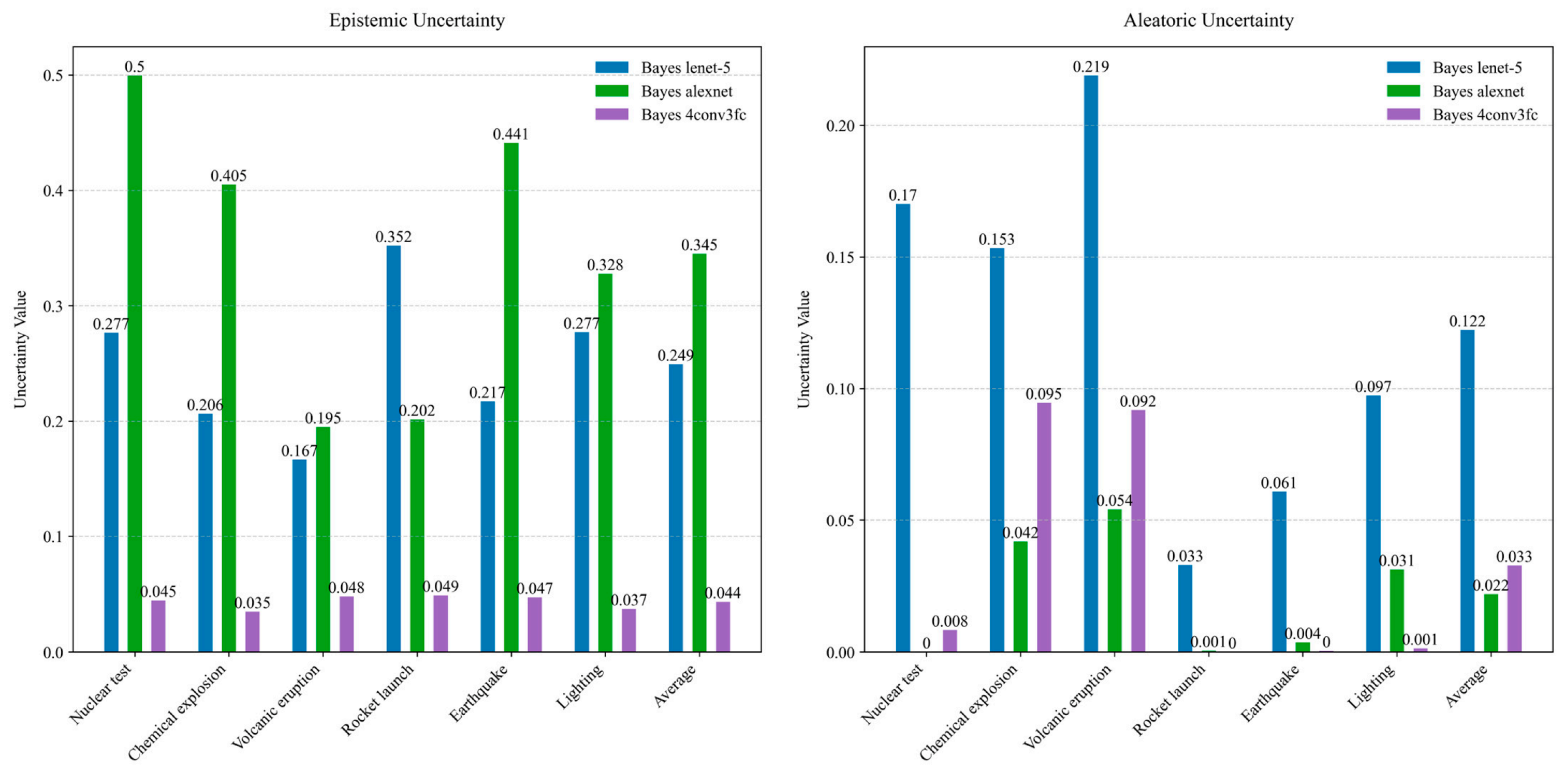
Uncertainty estimation of three Bayesian CNN.

As shown in Figure 10, the epistemic uncertainty of Bayes LeNet-5 is generally high, with an average value of 0.249, and the epistemic uncertainty of rocket launch signals is highest (0.352). This observation suggests that the shallow architecture of Bayes LeNet-5 struggles to capture the complex time–frequency features of infrasound events, particularly for classes with limited training samples. Its aleatoric uncertainty is also the highest among the three models, averaging 0.122, with volcanic eruptions exhibiting the largest value (0.219), reflecting the substantial intra-class variability inherent to volcanic activity rather than merely indicating the presence of noise.

Bayes AlexNet presents the highest average epistemic uncertainty (0.345), which we attribute to a mismatch between its model complexity and the available training data volume. As shown in Table 4 and Table 5, its ROC-AUC and PR-AUC performance is inferior to the other two models across most classes, especially for nuclear tests, chemical explosions, volcanic eruptions, and rocket launches. Notably, its aleatoric uncertainty is the lowest overall (average: 0.022), with slightly elevated values only for chemical explosions (0.042) and volcanic eruptions (0.054). This pattern—high epistemic uncertainty coupled with low aleatoric uncertainty—indicates that the predictive variability of Bayes AlexNet is dominated by insufficient knowledge of the parameters rather than by noise in the data, consistent with the behaviour of an over-parameterised model trained on a limited and imbalanced sample.

In contrast, Bayes 4Conv3Fc achieved the lowest epistemic uncertainty (average: 0.044), with all class-level values below 0.05, indicating that its architecture is well matched to both the data scale and the classification task. Its aleatoric uncertainty (average: 0.033) lies between that of Bayes AlexNet and Bayes LeNet-5. As with the other models, chemical explosions (0.095) and volcanic eruptions (0.092) exhibit relatively high aleatoric uncertainty, which, as discussed below, is more indicative of data complexity than of model deficiency.

Several noteworthy phenomena emerge from the cross-model comparison. Bayes LeNet-5 exhibits the highest epistemic uncertainty for rocket launch signals (0.352) but the lowest aleatoric uncertainty for this class (0.033), implying that although the data quality is adequate, the model lacks the capacity to learn the distinctive non-stationary patterns of rocket launch events. Bayes AlexNet, by contrast, shows an extremely high epistemic uncertainty (0.5) for nuclear test signals alongside near-zero aleatoric uncertainty, suggesting a fundamental difficulty in forming stable predictions for this rare class, likely due to the combined effect of model over-parameterization and insufficient training examples. Bayes 4Conv3Fc maintains consistently low epistemic uncertainty across all classes, yet the aleatoric uncertainty for chemical explosions and volcanic eruptions remains notably higher than for other events, reinforcing the interpretation that this elevation stems from intrinsic data properties rather than model limitations.

Analyzing the sources of uncertainty in conjunction with physical characteristics enables further diagnosis of model behavior and dataset quality. In the Bayes 4Conv3Fc model, the aleatoric uncertainty for chemical explosions and volcanic eruptions is considerably higher than that for other classes (0.095 and 0.092, respectively), which aligns closely with the intrinsic variability of these two event types: differences in yield, burst height, and charge type cause substantial variations in the source spectra of chemical explosions, while natural fluctuations in eruption intensity, column height, and duration lead to highly diverse spectrograms within the volcanic eruption class. Consequently, the elevated aleatoric uncertainty is not a deficiency of the model but a genuine reflection of the inherent complexity of the data, suggesting that future efforts should focus on collecting more diverse samples or establishing finer subcategories for these two event types. In contrast, nuclear tests and rocket launches exhibit extremely low aleatoric uncertainty, indicating that the acoustic signals of these events are relatively consistent within the given dataset and that the prediction variability for these classes mainly originates from epistemic uncertainty—for nuclear tests, this arises from insufficient model learning due to the limited number of samples, while for rocket launches, it is associated with the distinctive non-stationary structure of the signals, which demands greater feature extraction capability. Lightning and earthquake events both present low epistemic uncertainty, confirming that the model has adequately captured their short and stable time–frequency patterns, such as the low-frequency dominant peak of earthquakes and the impulsive broadband spectrum of lightning. Overall, uncertainty decomposition not only provides guidance for model optimization but also offers a quantitative perspective for understanding the physical observability of different infrasound sources.

While the above decomposition yields descriptive insights, the calibration of the resulting predictive distributions must be examined to verify that these uncertainty estimates are operationally meaningful. To this end, we compute the Brier score for both the class-weighted deterministic models and the Bayesian models. A lower Brier score indicates a smaller mean squared error between the predicted probabilities and the true class labels, reflecting better overall probabilistic calibration. The experimental results, reported in Table 11, show that across all three architectures, the Bayesian models consistently achieve lower Brier scores than their class-weighted deterministic counterparts. Specifically, the Bayesian models reduce the Brier score by approximately 40.6%, 42.0%, and 28.9%, respectively. Moreover, the standard deviations of the Bayesian models are markedly lower, demonstrating greater stability across different data splits.

These quantitative calibration results complement the uncertainty decomposition findings. The lower Brier score of Bayesian models confirms that their predictive probabilities are better aligned with the true outcomes, which means that the epistemic and aleatoric uncertainty estimates described above are not merely descriptive statistics but correlate with genuinely improved output reliability. The calibration gain for AlexNet after Bayesian treatment is remarkable. The AlexNet-weights achieved a poor Brier score of 0.162—the worst among all weighted models—while its Bayesian counterpart exhibited a high epistemic uncertainty of 0.345 and a much lower Brier score (0.094). This combined improvement reinforces the interpretation that Bayesian weight uncertainty modeling alleviates overconfidence while sharpening predictive distributions where data permit.. In contrast, the class-weighted cross-entropy approach mainly adjusts the loss contribution to mitigate imbalance; it does not guarantee calibrated probabilities, as evidenced by the higher Brier scores of the weighted models. Thus, the Brier score advantage of Bayesian models indicates that their performance gains cannot be fully attributed to class re-weighting or simple regularization but rather involve a principled integration of parameter uncertainty that benefits both classification accuracy and probability quality.

In summary, the proposed Bayesian CNN provide not only well-decomposed uncertainty estimates that diagnose model capacity and data complexity but also deliver superior probabilistic calibration as validated by the Brier score. These properties together make the Bayesian framework particularly suitable for infrasound monitoring tasks where trustworthy confidence measures are essential.

## 4. Conclusions

This paper proposes a Bayesian CNN framework for infrasound signal classification. The framework directly utilizes time–frequency spectrograms without any data augmentation. By placing probability distributions over the network weights and performing variational inference, the model can effectively learn from small-scale, highly imbalanced datasets while providing meaningful uncertainty estimates. The Bayes 4Conv3Fc model achieves an accuracy of 99.14% on the test set, with the F1-score improved by 2.83 percentage points compared with its deterministic counterpart. The classic AlexNet architecture benefits even more considerably, with Bayesian CNN improving its accuracy by 6.58 percentage points. Furthermore, when compared against class-weighted cross-entropy—a standard imbalance-handling baseline—the Bayesian models consistently yield substantially lower Brier scores (e.g., a 42.0% reduction for AlexNet), demonstrating superior probabilistic calibration. This confirms that the observed performance gains originate from principled uncertainty modeling rather than from mere loss re-weighting or ordinary regularization. The framework is model-agnostic and can be applied to existing CNN architectures with minimal modifications, making it a flexible basis for further exploration.

This study further decomposes the predictive uncertainty in infrasound signal classification into epistemic and aleatoric uncertainties. The calibration analysis validates that these uncertainty estimates are reliable, thereby enabling researchers to identify samples with low prediction confidence and to diagnose whether performance limitations originate from insufficient model capacity or data quality issues. Such diagnostic capability provides direct guidance for subsequent data acquisition or model improvement. By integrating uncertainty decomposition with calibration verification, the proposed method offers a foundation for developing high-accuracy, interpretable, and uncertainty-aware infrasound monitoring systems.

Future work will focus on lightweight Bayesian inference techniques to reduce training and inference time and optimize the number of Monte Carlo samples, with a complete computational cost analysis to be provided. Additional comparisons with focal loss, balanced sampling, Monte Carlo dropout, and deterministic ensembles, along with comprehensive calibration evaluations (e.g., reliability diagrams and expected calibration error), will be pursued to further isolate the unique contributions of Bayesian posterior marginalization.

## Figures and Tables

**Table 1:** Infrasound signal category and sample size.

Signal Category	Number of Events	Number of Samples
Nuclear tests	96	257
Chemical explosions	282	1084
Volcanic eruptions	86	543
Rocket launches	28	33
Earthquakes	19	137
Lightning	84	252
Total	595	2306

**Table 2:** The total number of layers of five classic convolutional neural network models.

Model	LeNet-5	AlexNet	VGGNet	GoogLeNet	ResNet
Total number of layers ^1^	5	8	16–19	22	152

^1.^ Layer counts include all convolutional, pooling, and fully connected layers. The 16–19 layers for VGGNet correspond to the VGG16 and VGG19 configurations, respectively.

**Table 3:** Subdataset partition scheme.

	Training Set		Verification Set		Test Set	
	Number of Events	Number of Samples	Number of Events	Number of Samples	Number of Events	Number of Samples
Nuclear tests	76	206	10	26	10	25
Chemical explosions	225	867	28	108	29	109
Volcanic eruptions	68	434	9	54	9	55
Rocket launches	21	25	3	4	4	4
Earthquakes	14	110	2	13	3	14
Lightning	67	201	8	25	9	26

**Table 4:** Performance comparison of the benchmark model and its Bayesian CNN.

Models	Accuracy	Precision	Recall	F1-Score	Cohen’s Kappa
LeNet-5	94.57%	94.61%	94.25%	94.25%	91.92%
Bayes LeNet-5	97.26%	97.32%	97.21%	97.19%	96.38%
AlexNet	92.25%	91.84%	91.59%	91.49%	89.59%
Bayes AlexNet	98.83%	98.75%	98.71%	98.69%	98.45%
4Conv3Fc	96.46%	96.37%	96.22%	96.22%	94.98%
Bayes 4Conv3Fc	99.14%	99.09%	99.05%	99.05%	98.98%

**Table 5:** ROC-AUC of multiple experiments.

Algorithms	LeNet-5	Bayes LeNet-5	AlexNet	Bayes AlexNet	4Conv3Fc	Bayes 4Conv3Fc
Nuclear test	0.985 ± 0.011	0.996 ± 0.005	0.985 ± 0.007	0.984 ± 0.018	0.993 ± 0.010	0.996 ± 0.008
Chemical explosion	0.993 ± 0.005	1 ± 0	0.99 ± 0	0.995 ± 0.005	0.997 ± 0.005	0.999 ± 0.003
Volcanic eruption	0.992 ± 0.004	0.999 ± 0.003	0.985 ± 0.005	0.992 ± 0.006	0.998 ± 0.004	0.997 ± 0.005
Rocket launch	0.996 ± 0.007	0.995 ± 0.016	0.953 ± 0.043	0.927 ± 0.086	1 ± 0	1 ± 0
Earthquake	0.997 ± 0.005	1 ± 0	0.992 ± 0.012	1 ± 0	1 ± 0	0.994 ± 0.014
Lightning	0.999 ± 0.003	1 ± 0	0.992 ± 0.012	1 ± 0	0.999 ± 0.003	0.997 ± 0.007

**Table 6:** PR-AUC of multiple experiments.

Algorithms	LeNet-5	Bayes LeNet-5	AlexNet	Bayes AlexNet	4Conv3Fc	Bayes 4Conv3Fc
Nuclear test	0.944 ± 0.026	0.984 ± 0.017	0.937 ± 0.023	0.974 ± 0.023	0.968 ± 0.024	0.988 ± 0.013
Chemical explosion	0.993 ± 0.005	1 ± 0	0.99 ± 0	0.998 ± 0.004	0.998 ± 0.004	0.999 ± 0.003
Volcanic eruption	0.972 ± 0.015	0.989 ± 0.011	0.945 ± 0.029	0.983 ± 0.010	0.991 ± 0.010	0.989 ± 0.007
Rocket launch	0.896 ± 0.166	0.965 ± 0.073	0.587 ± 0.197	0.927 ± 0.086	0.982 ± 0.041	0.994 ± 0.019
Earthquake	0.961 ± 0.030	0.99 ± 0.016	0.942 ± 0.044	0.997 ± 0.010	0.982 ± 0.011	0.983 ± 0.025
Lightning	0.983 ± 0.018	0.996 ± 0.007	0.977 ± 0.024	0.992 ± 0.010	0.991 ± 0.014	0.989 ± 0.019

**Table 7:** The index statistical results of 10-fold cross-validation.

Algorithms	LeNet-5	Bayes LeNet-5	AlexNet	Bayes AlexNet	4Conv3Fc	Bayes 4Conv3Fc
Accuracy	0.946 ± 0.014	0.973 ± 0.008	0.923 ± 0.015	0.988 ± 0.007	0.965 ± 0.016	0.991 ± 0.003
Precision	0.946 ± 0.012	0.973 ± 0.008	0.918 ± 0.016	0.988 ± 0.008	0.964 ± 0.015	0.991 ± 0.003
Recall	0.943 ± 0.013	0.972 ± 0.008	0.916 ± 0.015	0.987 ± 0.008	0.962 ± 0.016	0.991 ± 0.003
F1-Score	0.943 ± 0.013	0.972 ± 0.008	0.915 ± 0.014	0.987 ± 0.008	0.962 ± 0.016	0.991 ± 0.003
Cohen’s Kappa	0.919 ± 0.039	0.964 ± 0.023	0.896 ± 0.037	0.985 ± 0.013	0.950 ± 0.032	0.990 ± 0.009
ROC-AUC	0.992 ± 0.004	0.999 ± 0.003	0.984 ± 0.007	0.984 ± 0.018	0.999 ± 0.003	0.998 ± 0.004
PR-AUC	0.958 ± 0.026	0.988 ± 0.016	0.895 ± 0.038	0.980 ± 0.019	0.984 ± 0.011	0.990 ± 0.009

**Table 8:** Precision results for each category.

Algorithms	LeNet-5	LeNet-5-Weights	Bayes LeNet-5	AlexNet	AlexNet-Weights	Bayes AlexNet	4Conv3Fc	4Conv3Fc-Weights	Bayes 4Conv3Fc
Nuclear test	0.964 ± 0.034	0.967 ± 0.021	0.980 ± 0.019	0.901 ± 0.050	0.929 ± 0.032	0.981 ± 0.028	0.951 ± 0.032	0.942 ± 0.037	0.973 ± 0.031
Chemical explosion	0.971 ± 0.025	0.975 ± 0.019	0.986 ± 0.013	0.962 ± 0.018	0.970 ± 0.019	0.996 ± 0.005	0.982 ± 0.016	0.970 ± 0.023	0.996 ± 0.009
Volcanic eruption	0.900 ± 0.031	0.917 ± 0.035	0.953 ± 0.024	0.843 ± 0.026	0.845 ± 0.043	0.970 ± 0.020	0.952 ± 0.026	0.956 ± 0.027	0.979 ± 0.016
Rocket launch	0.932 ± 0.149	0.935 ± 0.106	0.920 ± 0.103	0.733 ± 0.306	0.758 ± 0.186	1 ± 0	0.927 ± 0.124	0.927 ± 0.124	1 ± 0
Earthquake	0.885 ± 0.107	0.927 ± 0.046	0.927 ± 0.044	0.891 ± 0.083	0.901 ± 0.055	0.993 ± 0.021	0.888 ± 0.091	0.909 ± 0.059	0.987 ± 0.028
Lightning	0.956 ± 0.043	0.956 ± 0.041	0.992 ± 0.016	0.957 ± 0.045	0.952 ± 0.034	0.985 ± 0.019	0.963 ± 0.045	0.961 ± 0.049	0.989 ± 0.019

**Table 9:** Recall results for each category.

Algorithms	LeNet-5	LeNet-5-Weights	Bayes LeNet-5	AlexNet	AlexNet-Weights	Bayes AlexNet	4Conv3Fc	4Conv3Fc-Weights	Bayes 4Conv3Fc
Nuclear test	0.836 ± 0.040	0.88 ± 0.0533	0.924 ± 0.034	0.856 ± 0.050	0.88 ± 0.0533	0.968 ± 0.037	0.916 ± 0.064	0.952 ± 0.032	0.984 ± 0.021
Chemical explosion	0.962 ± 0.019	0.973 ± 0.011	0.992 ± 0.008	0.943 ± 0.024	0.946 ± 0.016	0.988 ± 0.009	0.976 ± 0.017	0.984 ± 0.007	0.990 ± 0.010
Volcanic eruption	0.953 ± 0.020	0.964 ± 0.021	0.973 ± 0.028	0.936 ± 0.041	0.941 ± 0.037	0.995 ± 0.009	0.962 ± 0.029	0.98 ± 0.013	0.993 ± 0.013
Rocket launch	0.850 ± 0.175	0.875 ± 0.132	0.900 ± 0.174	0.525 ± 0.237	0.725 ± 0.185	0.850 ± 0.175	0.875 ± 0.213	0.925 ± 0.121	0.950 ± 0.105
Earthquake	0.943 ± 0.066	0.95 ± 0.048	0.972 ± 0.032	0.821 ± 0.103	0.886 ± 0.077	0.993 ± 0.023	0.936 ± 0.041	0.95 ± 0.035	0.979 ± 0.048
Lightning	0.954 ± 0.065	0.962 ± 0.054	0.946 ± 0.050	0.927 ± 0.055	0.954 ± 0.035	1 ± 0	0.970 ± 0.035	0.977 ± 0.027	0.985 ± 0.027

**Table 10:** F1-Score results for each category.

Algorithms	LeNet-5	LeNet-5-Weights	Bayes LeNet-5	AlexNet	AlexNet-Weights	Bayes AlexNet	4Conv3Fc	4Conv3Fc-Weights	Bayes 4Conv3Fc
Nuclear test	0.895 ± 0.028	0.922 ± 0.028	0.950 ± 0.017	0.877 ± 0.036	0.903 ± 0.035	0.974 ± 0.023	0.932 ± 0.041	0.951 ± 0.025	0.978 ± 0.020
Chemical explosion	0.966 ± 0.015	0.974 ± 0.011	0.989 ± 0.007	0.952 ± 0.016	0.957 ± 0.009	0.992 ± 0.004	0.979 ± 0.009	0.976 ± 0.014	0.993 ± 0.008
Volcanic eruption	0.925 ± 0.009	0.939 ± 0.021	0.962 ± 0.015	0.887 ± 0.026	0.889 ± 0.018	0.982 ± 0.010	0.957 ± 0.020	0.968 ± 0.017	0.986 ± 0.013
Rocket launch	0.872 ± 0.127	0.896 ± 0.082	0.894 ± 0.094	0.583 ± 0.243	0.723 ± 0.130	0.910 ± 0.111	0.887 ± 0.154	0.915 ± 0.077	0.971 ± 0.060
Earthquake	0.910 ± 0.072	0.937 ± 0.023	0.948 ± 0.036	0.850 ± 0.063	0.891 ± 0.053	0.993 ± 0.015	0.909 ± 0.053	0.928 ± 0.024	0.982 ± 0.027
Lightning	0.953 ± 0.039	0.958 ± 0.036	0.968 ± 0.032	0.941 ± 0.045	0.952 ± 0.019	0.993 ± 0.010	0.966 ± 0.033	0.968 ± 0.024	0.986 ± 0.021

**Table 11:** Comparison of Brier scores between the Bayesian CNN and the deterministic model with class-weighted cross-entropy loss.

Model	Brier Score
LeNet-5-weights	0.096 ± 0.032
Bayes LeNet-5	0.057 ± 0.005
AlexNet-weights	0.162 ± 0.027
Bayes AlexNet	0.094 ± 0.009
4Conv3Fc-weights	0.090 ± 0.024
Bayes 4Conv3Fc	0.064 ± 0.006

## Data Availability

The data and code that support the findings of this study are openly available from the corresponding author upon reasonable request, with the exception of the nuclear test data, which are subject to access restrictions.
